# Mouse superkiller‐2‐like helicase DDX60 is dispensable for type I IFN induction and immunity to multiple viruses

**DOI:** 10.1002/eji.201545794

**Published:** 2015-10-12

**Authors:** Delphine Goubau, Annemarthe G. van der Veen, Probir Chakravarty, Rongtuan Lin, Neil Rogers, Jan Rehwinkel, Safia Deddouche, Ian Rosewell, John Hiscott, Caetano Reis e Sousa

**Affiliations:** ^1^ Immunobiology LaboratoryThe Francis Crick InstituteLincoln's Inn Fields Laboratory44 Lincoln's Inn FieldsLondonUK; ^2^ BioinformaticsThe Francis Crick Institute, Lincoln's Inn Fields LaboratoryLondonUK; ^3^ Molecular Oncology GroupLady Davis Institute—Jewish General Hospital, McGill UniversityMontrealQuebecCanada; ^4^ Transgenic ServicesThe Francis Crick Institute, Clare Hall LaboratoryPotters BarHertsUK; ^5^ Vaccine & Gene Therapy Institute of FloridaPort Saint LucieFLUSA

**Keywords:** DDX60 ⋅ Innate immunity ⋅ Interferon ⋅ RIG‐I‐like helicases

## Abstract

IFN‐α/β allow cells to fight virus infection by inducing the expression of many genes that encode effectors of antiviral defense. One of these, the Ski2‐like DExH‐box helicase DDX60, was recently implicated in resistance of human cells to hepatitis C virus, as well as in induction of IFN‐α/β by retinoic acid inducible gene 1‐like receptors (RLRs) that detect the presence of RNA viruses in a cell‐intrinsic manner. Here, we sought to investigate the role of DDX60 in IFN‐α/β induction and in resistance to virus infection. Analysis of fibroblasts and myeloid cells from *Ddx60*‐deficient mice revealed no impairment in IFN‐α/β production in response to RLR agonists, RNA viruses, or other stimuli. Moreover, overexpression of DDX60 did not potentiate IFN induction and DDX60 did not interact with RLRs or capture RLR agonists from virally infected cells. We also failed to identify any impairment in Ddx60‐deficient murine cells or mice in resistance to infection with influenza A virus, encephalomyocarditis virus, Sindbis virus, vaccinia virus, or herpes simplex virus‐1. These results put in question the reported role of DDX60 as a broad‐acting positive regulator of RLR responses and hint at the possibility that it may function as a restriction factor highly specific for a particular virus or class of viruses.

## Introduction

Infection of cells by a virus triggers a potent and rapid cell‐intrinsic innate immune response centered around the expression of type I interferons (mainly IFN‐α/β) cytokines. The latter induce the expression of hundreds of IFN‐stimulated genes (ISGs) that contribute to viral clearance and favor the initiation of antiviral adaptive immune responses 1, 2, 3. IFN‐α/β genes are expressed in response to signals from pattern recognition receptors (PRRs) that are expressed in all nucleated cells and detect atypical nucleic acids associated with virus infection 1, 3. For RNA viruses, PRRs include the retinoic acid inducible gene 1 (RIG‐I)‐like DExD/H‐box RNA helicases (RLRs), RIG‐I, melanoma differentiation‐associated gene 5 (MDA5), and Laboratory of Genetics and Physiology 2 (LGP2), which survey the cytoplasm for the presence of RNAs with 5′ di‐ and triphosphate moieties and/or presenting distinct inter‐ or intramolecular base‐paired structures 1, 4. RLRs activated by such RNAs engage the adaptor mitochondrial antiviral‐signaling protein (MAVS) that coordinates the activation of IFN regulatory factor 3 (IRF‐3) and NF‐κB transcription factors to induce the IFN genes 1, 4. Infection by DNA viruses can also be indirectly sensed by RLRs, via a 5′ triphosphate‐containing RNA intermediate 5, 6, but in many instances is directly detected by PRRs that bind DNA 1. The latter include the recently identified cyclic GMP‐AMP synthase (cGAS) enzyme that produces in response to DNA stimulation a cyclic dinucleotide second messenger, which acts on a downstream adaptor known as stimulator of IFN gene (STING) that, like MAVS, activates IRF‐3 and NF‐κB to induce IFNs 7. Genetic ablation of RLRs, MAVS, cGAS, and STING in mice has underscored their critical role in immunity to different RNA and DNA viruses 8, 9, 10, 11, 12, 13, 14.

Like any other signaling pathway, the IFN induction axis is highly regulated, both negatively and positively, by accessory proteins and transcriptional and translational circuits 1, 2, 3. For example, RLRs are themselves ISGs, allowing for establishment of a potent positive feedback loop for IFN‐α/β induction 15. Other regulators of the IFN induction pathway and host defense are often IFN‐inducible and also include RNA helicases besides RLRs, such as DDX3, DHX9, DDX24, and the DDX1/DDX21/DHX36 complex 1, 16. In vitro experiments have implicated a number of these helicases in potentiating IFN‐α/β expression, either by directly sensing nucleic acids following viral infection and/or by interacting with canonical components of the IFN‐α/β induction pathway 1, 16. However, a role for most of these helicases in innate sensing in vivo has yet to be determined, partly due to embryonic lethality following genetic loss.

A few years ago, our attention was caught by the IFN‐inducible DExH‐box helicase, DDX60 (also known as DHX60) 17. At that time, DDX60 was an uncharacterized DExH helicase related to the yeast Ski2 helicase. Ski2 is a core component of the Ski complex involved in 3′ to 5′ RNA degradation by the exosome, but was originally identified for its ability to suppress the endogenous yeast RNA viruses L‐A, L‐BC, and 20S RNA, indicating a role for Ski2 in intrinsic virus resistance in some species 18, 19, 20. While our study of DDX60 was in progress or undergoing peer review, two reports were published by the Seya group that suggested that DDX60 is a broad‐acting potentiator of antiviral responses, binding to viral RNA (vRNA) and associating with RLRs to enhance signaling through MAVS 21, 22. A separate study did not find DDX60 to be broadly acting in antiviral immunity, but implicated it selectively in restriction of hepatitis C virus (HCV) replication, suggesting a virus‐specific defense role 23. Here, we used in vitro and in vivo approaches, including analysis of a *Ddx60*‐deficient mouse model, to find that murine DDX60 is dispensable for the induction of IFN‐α/β in response to different RLR agonists and for resistance to infection by multiple viruses. These results put in question the notion of DDX60 as a major component of IFN‐inducing RLR pathways across all cell types and underscore the possibility that DDX60 may have specialized functions that are only apparent in response to a subset of viruses or other pathogens.

## Results

### DDX60 is an IFN inducible Ski2‐like helicase

To identify helicases that might modulate RLR signaling, we screened microarray data for ones upregulated in primary human macrophages upon expression of constitutively active forms of IRF‐3 and IRF‐7, which induce type I IFN 17. *DDX60* expression was upregulated more than 2.4‐fold following IRF‐3 or IRF‐7 overexpression and was selected for further study.

**Figure 1:**
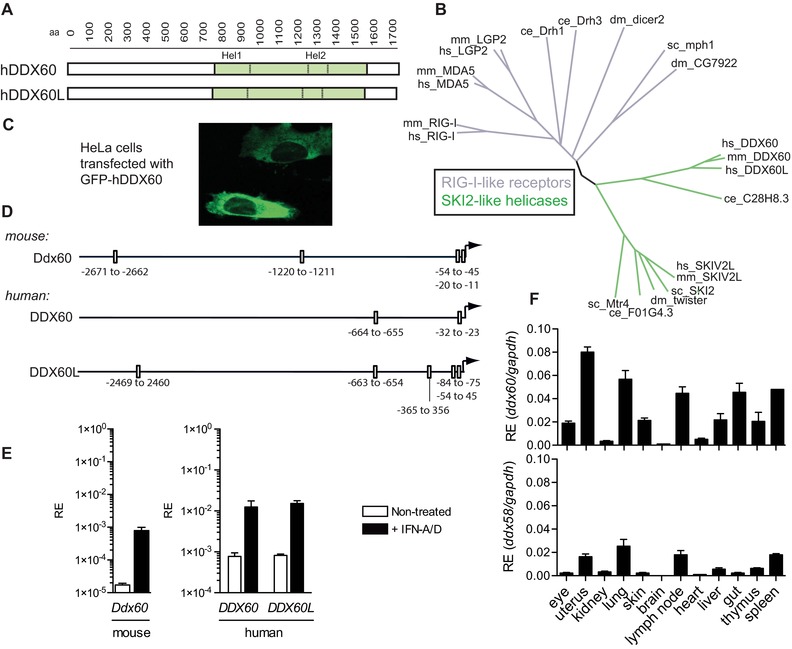
DDX60 and DDX60L are IFN inducible Ski2‐like DExH helicases. (A) Schematic representation of the human Ski2‐like helicases DDX60 and DDX60L highlighting the helicase domain. Hel1 and Hel2 denote the consensus subdomains of the helicase domain. (B) Phylogenetic relationship of the Hel1 helicase subdomain (containing the DExH/D Walker B motif) of RIG‐I‐like and Ski2‐like helicases from human (hs), mouse (mm), fly (*Drosophila melanogaster*, dm), yeast (*Saccharomyces cerevisae*, sc), and nematode (*Caenorhabditis elegans*, ce). (C) Microscopy image of live HeLa cells transfected with GFP‐tagged hDDX60. (D) Schematic representation of consensus ISRE binding sites found by computational analysis in the promoter of human and mouse *DDX60* and in human *DDX60L*. (E) Murine NIH3T3 (left panel) and human HEK293 cells (right panel) were treated or not with recombinant IFN (+IFN‐A/D) for 8 h. The relative expression (RE) of human and murine *DDX60* and human DDX60L was assessed by Q‐PCR and normalized to GAPDH. (F) RE of murine *Ddx60* and *Ddx58* (*Rig‐i*) from indicated murine organs. For PCR data, the mean (±SD) of triplicate technical replicates is shown. For experiments (C–E) one representative of three independent experimental repeats is shown, for (F) one of two is shown.

Human DDX60 (hDDX60) is 1712aa long and is not known to possess sequence features other than a helicase domain (761–1589aa), which has close homology to that of Ski2 helicases (Fig. 1A and B). Like Ski2, DDX60 is evolutionarily conserved and is found in mammals and in *Caenorhabditis elegans*. Unsurprisingly, phylogenetic comparison of helicase domains revealed that RLRs and DDX60 form distinct families across human, mouse, fly, yeast, and nematode (Fig. 1B). Interestingly, although DDX60 and Ski2 helicases belong to a common branch of Ski2‐like helicases, their sequences deviate enough from one another to form separate sub‐branches (Fig. 1B). This is further highlighted in the 23% identity of their Hel1 domain within the helicase core and 10% identity across their entire human sequences. In contrast, the RLR‐helicase family members cluster closer together (Fig. 1B). Thus, DDX60 constitutes a distinct member of the family of Ski2‐like helicases. Live imaging of HeLa cells expressing a GFP‐tagged version of hDDX60 shows that like RLRs and Ski2 20, DDX60 is cytosolic as previously reported (Fig. 1C) 21. Another helicase, annotated as DDX60‐like (DDX60L) in the National Center for Biotechnology Information (NCBI) database appears to be restricted to certain primates and horses and is not found in mice. In humans, the DDX60L gene is adjacent to that of DDX60 on chromosome 4 at position 4q32.3. This region appears to be syntenic with the mouse genome (chr8 qB3.1) according to NCBI's HomoloGene (http://www.ncbi.nlm.nih.gov/homologene). The order of human genes residing in this region (ANXA10, DDX60, DDX60L, and PALLD) appears to be conserved in the mouse, except for DDX60L, which is absent. Protein alignment studies demonstrate that hDDX60 and hDDX60L are highly homologous with a 65% identity score (Fig. 1A and B).

Analysis of 3 kB of sequence upstream of the start codon demonstrated that both human and mouse *DDX60* contain two to four IFN‐stimulated response elements (ISREs) within the *DDX60* promoter (Fig. 1D), validating their identification as ISGs. Corroborating this observation, quantitative (Q‐) PCR analysis revealed markedly increased expression of human and mouse DDX60 mRNA in type I IFN‐treated cells relative to controls (Fig. 1E) 21. The *DDX60L* promoter also contains ISREs and *DDX60L* mRNA is similarly IFN‐inducible (Fig. 1D and E). Thus, expression of both DDX60 and DDX60L can be induced upon exposure to type I IFNs. However, as DDX60L is not conserved in mice, we focused our subsequent analysis almost exclusively on DDX60.

Both BioGPS gene expression profiling [http://biogps.gnf.org] and levels of *Ddx60* mRNA from different murine organs (Fig. 1F) correlated with one another and demonstrated that Ddx60 is expressed in most tissues with the exception of the brain, kidney, and heart. The mRNA profiles of *Ddx60* and *Ddx58* (encoding RIG‐I) across different tissues were very similar (Fig. 1F). Comparable expression was also seen at a cellular level where *Ddx60* and *Ddx58* mRNAs appear present in most immune cells with the exception of certain dendritic cell subsets [http://www.immgen.org/index_content.html] 24.

### Overexpression of DDX60 does not potentiate IFN induction

**Figure 2:**
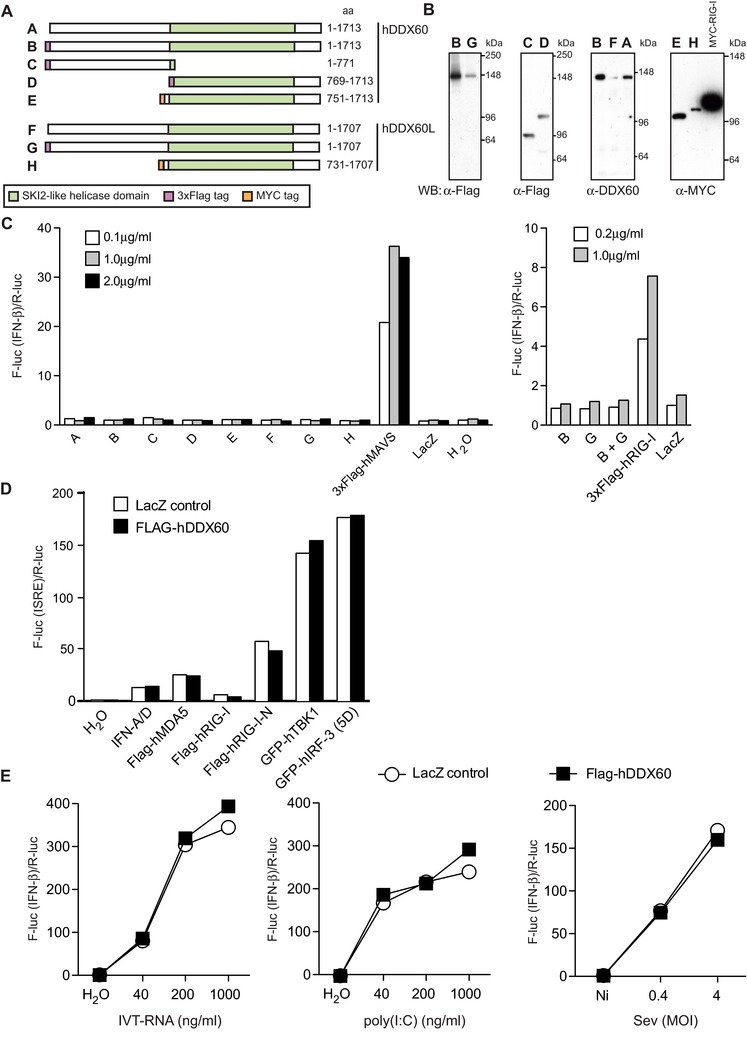
Overexpression of DDX60 or DDX60L does not induce type I IFNs. (A) Different human DDX60 and DDX60L constructs labeled A to H used in (B) for Western blot analysis and (C) IFN‐β promoter reporter assay. For (B), HEK293 cells were transfected with indicated plasmids and total cells lysates analyzed by Western blot. Membranes were probed with indicated antibodies. MYC‐hRIG‐I transfection was used as a control. In (C) HEK293 cells were cotransfected with an IFN‐β promoter firefly luciferase reporter, renilla luciferase control, and indicated amounts of hDDX60 and/or hDDX60L expression plasmids. Twenty‐four hours later, firefly luciferase (F‐luc) activity was measured and normalized to renilla luciferase (R‐luc) activity and to the water control. Plasmids coding 3xFlag‐hMAVS and 3xFlag‐hRIG‐I were used as positive controls, whereas LacZ was used as a negative one. (D) HEK293 cells were cotransfected with an ISRE promoter firefly luciferase reporter, a Renilla luciferase control, 200 ng/mL of control LacZ or 3xFlag‐hDDX60 expressing plasmid as well as indicated hRIG‐I, hMDA5, hTBK1, or hIRF‐3 expressing plasmids or treated with IFN‐A/D (1000 IU/mL). Twenty‐four hours later, luciferase activity was measured. (E) HEK293 cells were cotransfected with an IFN‐β promoter firefly luciferase reporter, a Renilla luciferase control, and 200 ng/mL of control LacZ or 3xFlag‐hDDX60 expressing plasmid. Twenty‐four hours later, cells were transfected with IVT‐RNA or poly(I:C) or infected with SeV. After overnight culture, luciferase activity was measured. For all experiments, one representative of three independent experimental repeats is shown.

To shed light on a possible function of DDX60 in antiviral immunity, we tested if overexpression of DDX60 could potentiate type I IFN production. As seen in Figure 2A to C, ectopic overexpression of hDDX60 in HEK293 cells did not activate an IFN‐β promoter luciferase reporter. This is in contrast to MAVS, which did so in a dose‐dependent fashion, as previously reported 25, 26, 27, 28. Lack of activation of the IFN‐β reporter following hDDX60 overexpression was also observed when truncated versions of the protein were expressed (N‐terminus alone or C‐terminal helicase alone) and was independent of the presence of different tags (no tag, 3xFlag tag, or MYC tag; Fig. 2A to C). Expression of hDDX60L alone or with hDDX60 also had no effect (Fig. 2A to C). Next, we investigated whether DDX60 overexpression could potentiate the response induced by activators of the IFN induction pathway. Human DDX60 was coexpressed with hMDA5, hRIG‐I, hTBK1, or the constitutively active forms of hRIG‐I (RIG‐I‐N 29) or hIRF‐3 (IRF‐3‐5D 30), all of which induce expression of IFN genes as assessed by an ISRE‐luciferase assay. As seen in Figure 2D, none of these proteins caused an increase in luciferase activity upon DDX60 overexpression. We also wondered whether ectopic expression of DDX60 could increase levels of IFN induced by RLR agonists or by virus infection. To this end, transiently transfected HEK293 cells expressing hDDX60 were stimulated with in vitro transcribed 5′ triphosphate‐containing RNA (IVT‐RNA) or poly(I:C) or were infected with Sendai virus (SeV), all of which trigger RLRs (Fig. 2E). However, overexpression of DDX60 did not increase the activity of the IFN‐β promoter in response to any of these three stimuli. Altogether, these data indicate that under these experimental conditions overexpression of DDX60 alone or in combination with DDX60L or other activators of the RLR pathway does not potentiate IFN induction.

### Generation of a Ddx60‐deficient mouse model

In order to address the role of DDX60 in antiviral defense in loss‐of‐function experiments, we set out to generate a Ddx60‐deficient (KO) mouse strain using *Ddx60*‐targeted C57BL/6 embryonic stem (ES) cells from the European Conditional Mouse Mutagenesis Program (EUCOMM) 31. The ES cells used here are incidentally the same ones used to generate the other very recently reported DDX60 KO mice 22. The targeting construct generates a “KO‐first” *lacZ*‐reporter conditional allele (Supporting Information Fig. 1A) through splicing of exon 8 to a *lacZ* element contained within the targeting cassette. Both the splice acceptor site (En2 SA) and the SV40 polyadenylation site (pA) are predicted to facilitate this event while an internal ribosomal entry site enables the translation of the *lacZ* reporter. Crosses to “flippase” (FLP) mice recombines the sequences between the flippase recognition target (FRT) sites and can revert the mutant allele back to a WT one where exon 9 (termed the “critical” exon by EUCOMM) is now flanked by *loxP* sites and can therefore be excised when crossed to Cre recombinase expressing strains. Deletion of exon 9 by Cre‐mediated recombination and splicing of exon 8–10 introduces a frameshift, resulting in a STOP codon within exon 10. This truncates the mDDX60 protein and is also predicted to induce the nonsense‐mediated decay of the mutated transcripts.

We obtained two EUCOMM PCR‐validated ES cell clones termed A05 and D07, but only A05 showed correct targeting by Southern blot analysis using a neomycin‐specific probe (Supporting Information Fig. 1B). This clone was used for injection into C57BL/6 host blastocysts to generate chimeras that successfully transmitted to the germline. Correct targeting in *Ddx60^−/−^* mice was validated by PCR and Southern blot analysis (Supporting Information Fig. 1C and D). *Ddx60^+/−^* and *Ddx60^−/−^* mice were viable and heterozygote crosses produced offspring at normal Mendelian ratios (Supporting Information Fig. 1E). No abnormalities were reported following full histopathological examination of 8‐ to 10‐week‐old male or female mice (data not shown).

**Figure 3:**
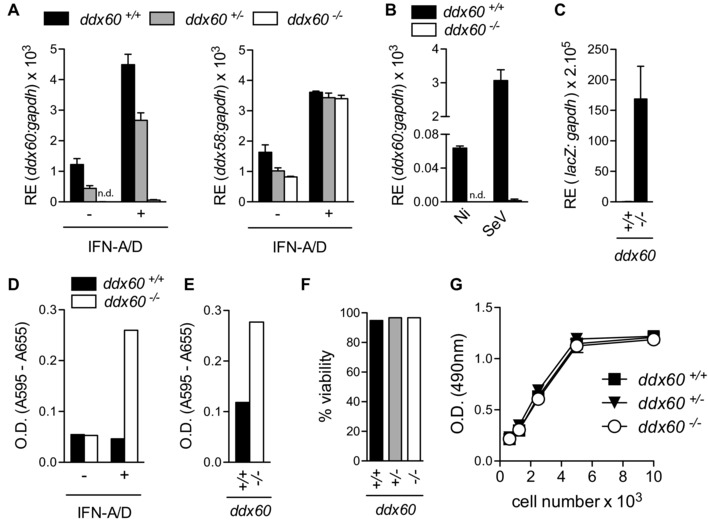
Validation of *Ddx60* KO mouse. (A) Expression *Ddx60* and *Ddx58* (*rig‐i*) from *Ddx60^+/+^*, *Ddx60^+/−^*, and *Ddx60^−/−^* MEFs treated (+) or not (−) with recombinant IFN‐A/D (16 h, 1000 IU/mL) (B) Expression of *Ddx60* from *Ddx60^+/+^* and *Ddx60^−/−^* BMMCs infected with SeV (multiplicity of infection 20) for 6 h. (C) Expression *lacZ* mRNA from large‐T antigen transformed *Ddx60^+/+^* or *Ddx60^−/−^* MEFs treated with IFN‐A/D (16 h, 1000 IU/mL). (D) β‐Galactosidase activity from 1 × 10^6^ LT‐transformed *Ddx60^+/+^* or *Ddx60^−/−^* MEFs treated (+) or not (−) with recombinant IFN‐A/D (1000 IU/mL; 16 h). (E) β‐Galactosidase activity from 1 × 10^6^ total splenocytes from *Ddx60^+/+^* or *Ddx60^−/−^* mice treated overnight with poly(I:C) (i.p., 200 μg/mouse). (F) Data from Trypan blue exclusion assay from MEFs. Data represents the average from at least two different embryos per genotype. (G) Indicated numbers of MEFs were plated and cell proliferation was assessed 5 days later. Each data point represents the mean of eight wells. (n.d.: not detected). For PCR data, the mean (±SD) of triplicate technical replicates is shown. For all experiments one representative of three independent experimental repeats is shown.

Commercially available or in‐house–generated anti‐Ddx60 antibodies did not detect endogenous murine Ddx60 by immunoblot (data not shown). To examine the levels of Ddx60 in the “KO‐first” mice, we therefore resorted to the analysis of *Ddx60* mRNA amplicons immediately 3′ of the targeting cassette. These amplicons were undetectable in *Ddx60^−/−^* mouse embryonic fibroblasts (MEFs) and were diminished in heterozygote animals, while *Ddx58* transcripts, as a control, were unaffected (Fig. 3A). Similar data were obtained for myeloid cells derived from bone marrow (BM) cells cultured with GM‐CSF (BM‐derived myeloid cells; BMMCs; Fig. 3B). As expected, stimulation of MEFs with recombinant IFN (IFN‐A/D) or infection of BMMCs with SeV increased *Ddx60* expression in *Ddx60^+/+^* and *Ddx60^+/−^* cells (Fig. 3A and B). Q‐PCR values obtained in IFN‐stimulated *Ddx60^−/−^* cells corresponded to 0.2–3% of those obtained in nontreated WT cells, indicating that the cassette may allow only a minimal degree of normal mRNA splicing. L*acZ* transcripts were detectable in *Ddx60^−/−^* but not WT MEFs treated with IFN and this correlated with β‐galactosidase activity detected in vitro and in vivo (Fig. 3C to E). Ddx*60^−/−^* MEFs showed no defects in proliferation or viability (Fig. 3F and G). These data indicate that the “KO‐first” targeting cassette functioned as intended and produced a *Ddx60* reporter‐KO mouse, which is viable and fertile.

### IFN‐α/β induction is unaffected in *Ddx60^−/−^* murine cells in response to different RNA stimuli

**Figure 4:**
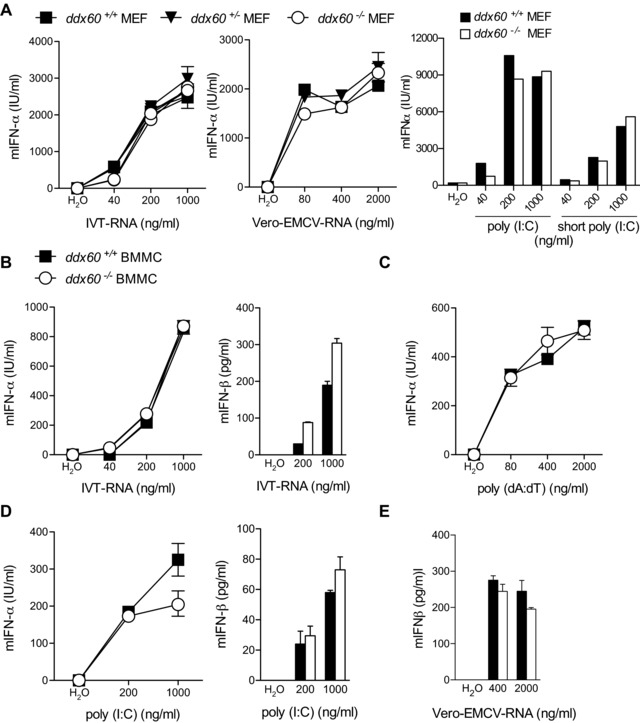
IFN‐α/β induction is unaffected in *Ddx60^−/−^* MEF and BMMCs in response to the transfection of different RNA stimuli. (A) Levels of mIFN‐α in 24 h culture supernatants from *Ddx60^+/+^*, *Ddx60^+/−^*, and *Ddx60^−/−^* MEFs transfected with indicated amounts of IVT‐RNA, poly(I:C), short poly(I:C), and RNA isolated from EMCV infected Vero cells (Vero‐EMCV‐RNA). (B–E) Levels of mIFN‐α or mIFN‐β in 24 h culture supernatants from *Ddx60^+/+^* and *Ddx60^−/−^* BMMCs transfected with different concentrations of (B) IVT‐RNA, (C) poly(dA:dT), (D) poly(I:C) or (E) Vero‐EMCV‐RNA. Water was used as a transfection control. The mean (±SD) of triplicate technical replicates is shown. *ns* refers to not significant. For all experiments one representative of three independent experimental repeats is shown.

**Figure 5:**
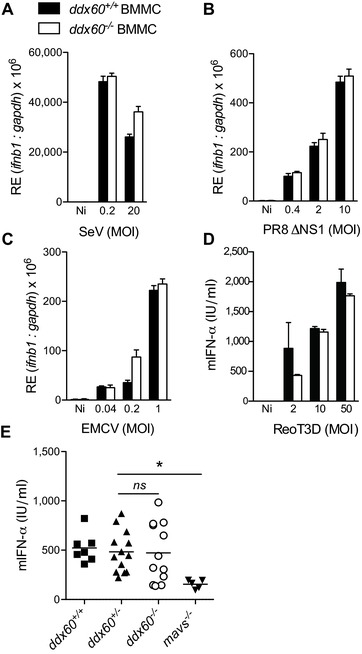
Ddx60 deficiency does not affect the induction of IFN‐α/β following infection with different RNA viruses. (A–C) Murine *ifnb1* expression from *Ddx60^+/+^* and *Ddx60^−/−^* BMMCs 6 h postinfection with (A) SeV, (B) influenza (PR8ΔNS1), (C) EMCV at indicated MOI. (D) Levels of mIFN‐α in 24 h culture supernatants from *Ddx60^+/+^* and *Ddx60^−/−^* BMMCs infected with ReoT3D at indicated MOIs. Noninfected (Ni) cells were also included. For (A–D) the mean (±SD) of triplicate technical replicates is shown. (E) Serum mIFN‐α levels from *Ddx60^+/+^*, *Ddx60^+/−^*, *Ddx60^−/−^*, and *mavs^−/−^* mice infected intraperitoneally with 1 × 10^6^ PFU of EMCV for 24 h. (*p = 0.004; Student's unpaired *t*‐test; *ns* refers to not significant with *p* = 0.7). Each symbol represents an individual mouse. Line represents the mean of each group. For experiments A–D one representative of three independent experimental repeats is shown. For E, the pooled results of three independent experiments are shown.

To determine a possible role for endogenous Ddx60 in type I IFN induction, as suggested 21, 22, we measured the response of “KO‐first” Ddx60‐sufficient and Ddx60‐deficient cells to IVT‐RNA, poly(I:C), poly(deoxyadenylic‐deoxythymidylic) acid sodium salt (poly(dA:dT)), or RNA extracted from encephalomyocarditis virus (EMCV) infected Vero cells (Vero‐EMCV‐RNA). Poly(dA:dT) is a form of B‐DNA that can be transcribed by RNA polymerase‐III into uncapped 5′ triphosphate‐bearing RNA that functions as a RIG‐I agonist 5, 6. Vero‐EMCV‐RNA is an MDA5 agonist while poly(I:C) can trigger both MDA5 and RIG‐I [12, 32]. In both MEFs and BMMCs, we observed no change in the levels of IFN‐α or IFN‐β associated with the lack of Ddx60 expression in response to any of the four stimuli (Fig. 4). Similarly, DDX60 deficiency did not impact the response to short poly(I:C) (Fig. 4A), a form of the polymer that is thought to preferentially trigger RIG‐I, in part due to the presence of 5′ di‐phosphates 33. Unaltered levels of *Ifnb1* transcripts were also observed in *Ddx60^+/+^* and *Ddx60^−/−^* BMMCs infected with different RNA viruses, including SeV, influenza A virus (IAV) lacking the NS1 protein (A/Puerto Rico/8/1934 H1N1 ΔNS1 abbreviated PR8 ΔNS1), reovirus (type 3 Dearing, reoT3D), and EMCV (Fig. 5A to D). Similarly, we observed no differences in serum IFN‐α levels between Ddx60‐sufficient and Ddx60‐deficient mice infected intraperitoneally with EMCV (Fig. 5E). *Mavs^−/−^* mice, used as positive controls, displayed a severely compromised response, as previously reported 14.

To further validate these results, we generated Ddx60 “full KO” mice, which no longer have the targeting cassette and where exon 9 was subsequently deleted by crossing to a line ubiquitously expressing Cre (Supporting Information Fig. 2A to D). Residual DDX60 transcripts were detected by RT‐qPCR in DDX60 “full KO” cells, but these are presumably degraded by nonsense‐mediated RNA decay as observed in Supporting Information Fig. 2C. Importantly, no truncated DDX60 ORF was detected in DDX60 “full KO” cells (Supporting Information Fig. 2D). As for cells derived from Ddx60 “KO first” mice, induction of IFN‐α was unaltered in BMMCs from Ddx60 “full KO” mice (Supporting Information Fig. 3A to C). This was unlike Mavs‐deficient cells, for which no IFN was detectable in response to any RLR agonists (Supporting Information Fig. 3A to C).

### DDX60 does not precipitate with RLRs or with stimulatory RNA

**Figure 6:**
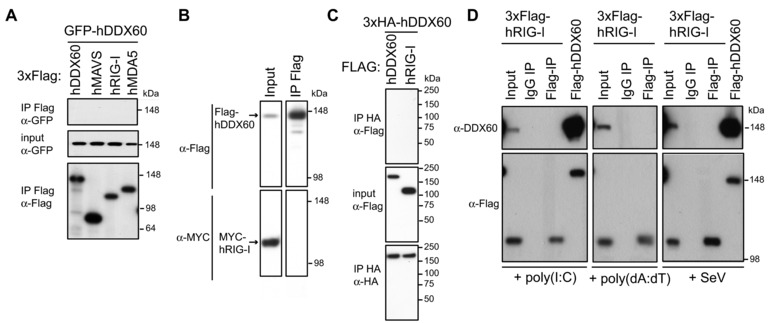
DDX60 does not precipitate with RIG‐I or MDA5. Western blot analyses were conducted on the following immunoprecipitates using indicated antibodies: (A) Flag precipitates from total cell lysates of HEK293T cells cotransfected for 24 h with GFP‐hDDX60 and 3xFlag‐tagged hMAVS, hRIG‐I, or hMDA5. (B) Flag precipitate from a total cell lysate of HEK293 cells stably expressing Flag‐hDDX60 transfected for 24 h with a MYC‐hRIG‐I expression plasmid. (C) HA precipitates from total cell lysates of HEK293T cells cotransfected for 48 h with 3xHA‐hDDX60 and 3xFlag‐tagged hDDX60 or hRIG‐I. (D) Flag and control IgG precipitates from total cell lysates of HEK293 cells stably expressing Flag‐hRIG‐I transfected with poly(I:C) or poly(dA:dT) or infected with SeV (MOI 1, 16 h). Total cell lysate from HEK293 cells stably expressing Flag‐hDDX60 were also included as a control. In (A–D) input samples (10% of total lysates before IP) were also analyzed. For experiments (A–C) one representative of three independent experimental repeats is shown, for (D) one of two is shown.

Given the reported interaction between DDX60, RLRs, and nucleic acids 21, 22, we sought to determine if DDX60 could interact with members of the cytosolic vRNA sensing pathway. GFP‐tagged hDDX60 was expressed in HEK293T cells together with Flag‐tagged hRIG‐I, hMDA5, hMAVS, or hDDX60, and possible interactions tested by immunoprecipitation (IP) with α‐Flag antibody followed by immunoblotting for GFP (Fig. 6A). No GFP‐tagged hDDX60 was found in Flag precipitates (Fig. 6A). To strengthen our analysis, we used cells transiently or stably expressing different tagged versions of hDDX60 or hRIG‐I, tested different antibodies for IP and detection, as well as tried milder lysis and precipitation conditions (0.5% NP40 buffer A, as detailed in Materials and methods). In no circumstances did we observe co‐IP of hDDX60 and hRIG‐I (Fig. 6B and C). We also generated, purified, and validated a polyclonal α‐DDX60 antibody (as detailed in Materials and methods) that allows for the detection of endogenous human (but not murine) DDX60. Although endogenous hDDX60 was clearly detectable in input material, it was absent in α‐Flag precipitates from HEK293 cells stably expressing Flag‐hRIG‐I and treated with different RLR‐agonists (poly(I:C), poly(dA:dT) or infected with SeV (Fig. 6D).

**Figure 7:**
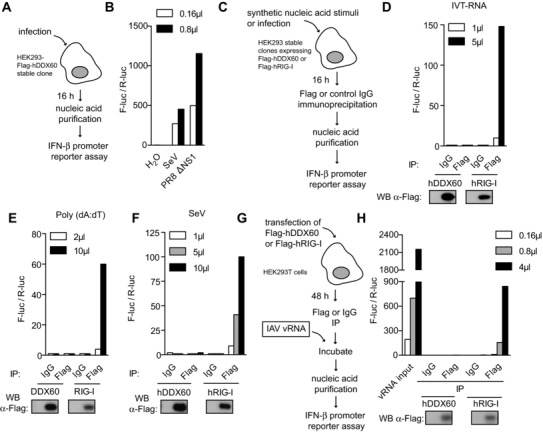
IP of DDX60 does not coprecipitate stimulatory RNA. (A) Diagram of experiment conducted in (B) where the stimulatory capacity of nucleic acid purified from stable HEK293‐Flag‐hDDX60 cells infected with SeV or PR8ΔNS1 (MOI 1) was assessed by IFN‐β promoter reporter assay. Water was used as a control. (C) Diagram of experiment conducted in (D, E, and F) where Flag‐hDDX60‐stable HEK293 cells were transfected with (D) IVT‐RNA or (E) poly(dA:dT) or infected with (F) SeV. Sixteen hours later, Flag‐hDDX60 was immunoprecipitated with an anti‐Flag antibody. An isotype‐matched IgG IP was included as control. RNA was then purified from precipitates and stimulatory capacity was assessed by IFN‐β promoter reporter assay (above). IP samples were also tested for Flag expression by Western blot (below). Experiment was conducted in conjunction with HEK293‐Flag‐hRIG‐I cells. (G) Diagram of experiment conducted in (H) where HEK293T cells were transfected with Flag‐hDDX60. Forty‐eight hours later, Flag‐hDDX60 was immunoprecipitated with an anti‐Flag antibody and an isotype‐matched IgG IP was included as control. Flag‐hDDX60‐coated IP beads as well as control beads were incubated with RNA isolated from IAV (IAV vRNA). Following extensive washes, RNA was purified from precipitates and stimulatory capacity was assessed by IFN‐β promoter reporter assay. IP samples were tested for Flag expression by Western blot. The experiment was conducted in conjunction with Flag‐hRIG‐I transfection. For all experiments one representative of three independent experimental repeats is shown.

Next, we sought to determine whether DDX60 could capture RLR agonists from virally infected cells. We analyzed the stimulatory capacity of RNA bound to full length Flag‐tagged hDDX60 precipitated from cells transfected with IVT‐RNA, poly(dA:dT), or infected with SeV virus (Fig. 7C to F). Unlike RIG‐I, used as a positive control 34, hDDX60 did not associate with stimulatory RNA in any conditions. This result could not be explained by the absence of stimulatory RNA, which was clearly present in HEK293 cells stably expressing Flag‐hDDX60 following infection (Fig. 7A and B). The absence of association between DDX60 and stimulatory RNA was also observed when IAV genomic RNA (vRNA) was mixed with Flag hDDX60 coated beads (Fig. 7G and H). Thus, under these experimental settings, we are unable to find evidence that DDX60 associates with RLRs or can capture RLR agonists.

### Absence of *Ddx60* does not affect the cytokine response to cytosolic DNA stimuli or TLR agonists

We also sought to determine whether the response induced via cGAS by DNA might be affected in *Ddx60^−/−^* cells. BMMCs were transfected with different amounts of DNA purified from *Escherichia coli* or calf thymus and the concentration of mIFN‐α in culture supernatants was measured by ELISA (Supporting Information Fig. 4A and B). Both *Ddx60^+/+^* and *Ddx60^−/−^* cells produced comparable amounts of IFN‐α in response to these stimuli. *Ifnb1* transcripts were also comparable in Ddx60‐sufficient and Ddx60‐deficient cells infected with the attenuated vaccinia virus (VACV), modified vaccinia Ankara (MVA; Supporting Information Fig. 4D). We also stimulated WT and Ddx60‐KO BMMCs with a cyclic‐dinucleotide (cyclic‐diguanylate monophosphate) that directly triggers STING 35, but found no difference in the magnitude of the IFN‐α response (Supporting Information Fig. 4E). This was irrespective of whether the cells were taken from Ddx60 “KO first” or Ddx60 “full KO” mice (Supporting Information Fig. 3D to F).

As the toll‐like receptor (TLR) pathway is another trigger of innate immune responses 36, *Ddx60^+/+^* and *Ddx60^−/−^* BMMCs were also tested for responsiveness to different TLR agonists, including LPS (TLR4), oligonucleotides containing unmethylated CG motifs (CpG; TLR9), and Pam3CSK4 (TLR2). As seen in Supporting Information Figure 5A to D, *Ifnb1* mRNA and IL‐6 protein levels were similar for both genotypes in response to all stimuli. The β‐(1,3)‐glucan polymer, curdlan, which acts as a selective Dectin‐1 agonist 37 and TNF‐α were also included in these analyses but, again, no alteration in IL‐6 induction was observed in *Ddx60*‐deficient cells (Supporting Information Fig. 5E and F). Altogether, these data reveal that DDX60 is dispensable for the induction of cytokines in response to agonists of the cytosolic DNA sensing pathway, of many TLRs, Dectin‐1 and the TNF receptor.

### Susceptibility to IAV, EMCV, Sindbis virus (SINV), *Listeria monocytogenes*, and HSV‐1 is unaltered by Ddx60 deficiency

**Figure 8:**
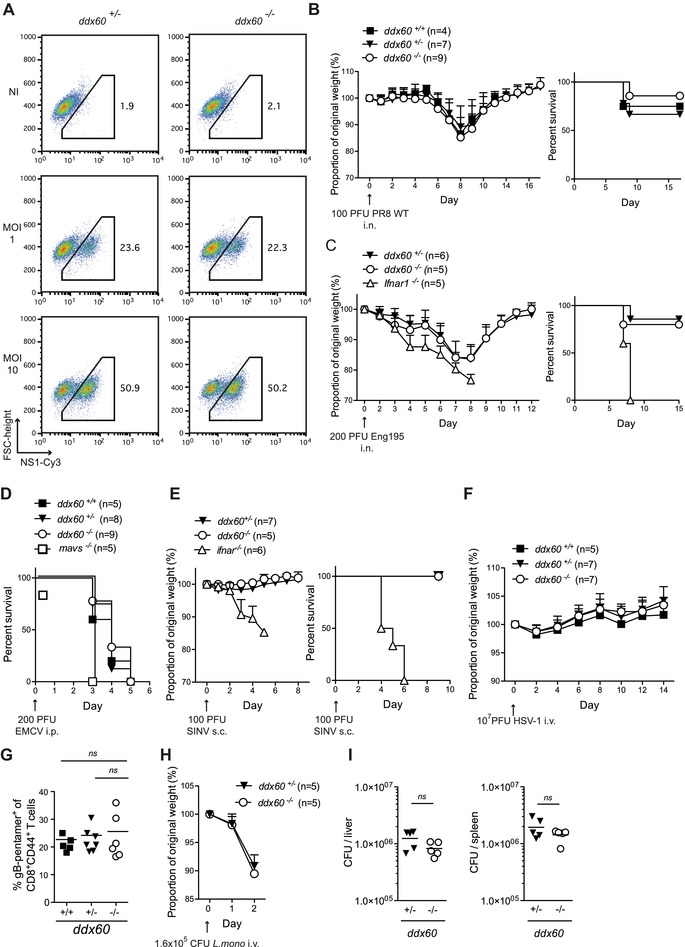
Ddx60 deficiency does not increase susceptibility to IAV, EMCV, SINV, HSV‐1, or *Listeria monocytogenes* infections. (A) MEFs of indicated genotypes were infected with IAV (PR8 WT) at an MOI of 1 or 10. Twenty‐four hours later, cells were harvested, fixed, and stained for NS1 using Cy3‐coupled anti‐NS1 antibody. Samples were then analyzed by FACS. Noninfected (Ni) cells were included as controls. Numbers represent the percentage of cells within the indicated gate. (B) Mice of specified genotypes were infected intranasally (i.n.) with 100 PFU of influenza virus (PR8 WT). The weight of each mouse was monitored daily and the proportion of weight loss from the day of the infection was calculated. Left panel shows the mean proportion of weight loss for all genotypes (±SD), whereas the right panel shows the percent survival. (C) Mice of specified genotypes were infected i.n. with 200 PFU of pandemic influenza virus (Eng195) and analyzed as in (B). (D) Survival curve of mice infected intraperitoneally with 200 PFU of EMCV. Graph shows pooled data from two independent experiments. (E) Mice were injected subcutaneously with 100 PFU of SINV and monitored as in (B). (F) Mice were injected i.v. with 10^7^ PFU of HSV‐1 and weight loss of each mouse was monitored as in (B). (G) Mice from (F) were bled on day 8 and the H‐2Kb‐gB pentamer positive (+) cells as a percentage of CD8^+^CD44^+^ T lymphocytes was quantified. (H) Mice were infected i.v. with 1.6 × 10^6^ CFU of *L. monocytogenes* and the weight of each mouse was monitored as in (B). (I) Mice in (H) were sacrificed and *L. monocytogenes* burden in their liver and their spleen was determined. Data shows the number of CFUs per organ for each mouse and the mean. *ns* refers to not statistically significant.

The demonstration that ectopic expression of DDX60 can restrict HCV replication 23 suggests that this helicase may play an important role in limiting viral spread. To investigate whether DDX60 might be necessary for host defense from infection despite its lack of involvement in IFN induction, we turned to a select number of murine viral infection models. All experiments were performed using Ddx60 “KO first” mice and cells, which were readily available. First, the potential role of Ddx60 in restricting IAV infections was assessed in vitro by measuring the amount of the influenza protein NS1 in *Ddx60^+/+^*, *Ddx60^+/−^*, and *Ddx60^−/−^* MEFs infected with IAV (A/Puerto Rico/8/1934 H1N1, abbreviated PR8 WT). As seen in Figure 8A, although there was a clear difference in the levels of NS1 between cells infected with two distinct MOIs, no difference was observed between genotypes. The unaltered sensitivity of *Ddx60^−/−^* cells to PR8 WT was recapitulated in vivo where no increase in weight loss or death was observed in *Ddx60^−/−^* mice relative to controls following intranasal infection (Fig. 8B). As PR8 WT is very virulent in laboratory mouse strains and may mask resistance attributable to IFN receptor signaling and ISGs 38, 39, 40, the experiments were repeated with a human isolate of the pandemic influenza A H1N1/09 Eng/195 (Eng195). This strain can be restricted in vivo by the ISG, IFN‐induced transmembrane protein 3 40 indicating that it is susceptible to IFN‐dependent control. The IFN‐susceptibility of Eng195 was confirmed by the observation that, unlike WT controls, all mice lacking the IFN‐receptor (*Ifnar^−/−^*) succumbed 7 days postinfection (Fig. 8C). In contrast, *Ddx60^−/−^* mice behaved like *Ddx60^+/+^* mice and lost weight as a result of the infection, but recovered within 11 days post infection (p.i.) (Fig. 8C). Thus, Ddx60 appears to be dispensable for mouse defense from experimental influenza virus infection.

Next, we tested the importance of Ddx60 in restricting two positive‐stranded strain RNA viruses, EMCV and SINV. Though the main reservoir host for EMCV is the rat, WT mice are very sensitive to the virus, even at low inoculum levels (41 and Fig. 8D). Innate immune pathways play a protective role as mice deficient in IFN or ISG induction, such as *Ifih1^−/−^* (MDA5 “KO”), *Mavs^−/−^*, or *Ifnar^−/−^* mice display an even greater susceptibility to this virus 12, 14, 42, and Fig. 8D). However, when *Ddx60^−/−^* mice were monitored following intraperitoneal (i.p.) infection with 200 plaque‐forming units (PFUs) of EMCV, no difference in mortality was observed relative to control WT or heterozygote groups (Fig. 8D). In contrast to EMCV infections, adult WT mice can easily resist SINV infections (43 and Fig. 8E). Mice with compromised innate host‐defense pathways, such as *Ifnar^−/−^* mice, start losing weight 3 days p.i. and die 1–3 days later ([43] and Fig. 8E). *Ddx60^−/−^* mice behaved like control mice and did not show any increased weight loss or susceptibility to SINV infection. Thus, the overall resistance to both EMCV and SINV infection appears unaltered in mice lacking Ddx60 expression.

We also evaluated the importance of Ddx60 in restricting two DNA viruses, VACV, and HSV‐1. We used two engineered VACV strains (the Western Reserve [WR] strain and the MVA strain) that express a red fluorescent protein (RFP) tagged version of the A3L gene, which encodes a major structural component of VACV virions 44. Cellular levels of A3L‐RFP were monitored by flow cytometry after 18 h of infection but no differences were seen between cells devoid or not of Ddx60 expression (Supporting Information Fig. 6). HSV‐1, unlike VACV, replicates in the nucleus and mice lacking cGAS or STING are susceptible to HSV‐1 infection 8, 10. However, *Ddx60^−/−^*, like *Ddx60^+/−^* and *Ddx60^+/+^*, mice did not succumb or lose any weight in response to the virus (Fig. 8F). At sacrifice, they also displayed equivalent percentages of CD8^+^CD44^+^ T cells specific for the viral glycoprotein B (gB), the immune‐dominant HSV‐1 epitope in C57BL/6 mice 45 (Fig. 8G), suggesting that Ddx60 deficiency does not impact at least some aspects of adaptive immunity to HSV‐1.

Lastly, as IFNs can play a role in bacterial as well as viral infections 46, we tested the susceptibility of *Ddx60*‐deficient mice to *L. monocytogenes* infection. Mice lacking the type I IFN receptor are resistant to this bacterium 47, 48. Both *Ddx60^+/−^* and *^−/−^* mice had parallel weight loss curves following infection (Fig. 8H) and titers of *L. monocytogenes* in the liver and the spleen, the primary sites of infection, were analogous in both groups (Fig. 8I). In sum, mouse DDX60 is dispensable for resistance to a wide range of experimental infections.

## Discussion

IFN‐α/β are major contributors to antiviral defense, inducing the expression of hundreds of ISGs that encode antiviral proteins as well as modulators of the IFN induction and response pathways. Here, we sought to further characterize the function of one of these ISGs, the DExH‐box helicase DDX60. Notably, two recent papers from the same group described DDX60 as an accessory protein in RLR signaling 21, 22. In the first manuscript, the authors suggested that DDX60 binds to RNA and DNA and identified RIG‐I, MDA5, LGP2, but not MAVS or IKKε as DDX60‐interacting partners. The authors further show that overexpression of DDX60 restricts both poliovirus and vesicular stomatitis virus replication. Finally, HeLa cell clones in which DDX60 expression was stably knocked‐down using shRNAs were shown to produce lower levels of IFN‐β and ISGs mRNA in response to poly(I:C), vesicular stomatitis virus, poliovirus, SeV, as well as HSV‐1. As DDX60 expression also appeared to increase binding of RNA to RIG‐I, the authors hypothesized that this helicase may bind vRNA and associate with RLRs during viral infections to enhance signaling 21. In their follow‐up study (published while this manuscript was undergoing peer review), the authors generated DDX60‐KO mice, using, as we did, EUCOMM targeted ES cells. They found that DDX60‐deficient MEFs, peritoneal macrophages, and splenic CD11c^+^ cells, but not BM‐derived DCs produced lower levels of type I IFN in response to stimulation when compared to controls. Despite the evidence for a function of DDX60 in RLR signaling emanating from those two studies, we did not observe any association of tagged or endogenous DDX60 with RIG‐I or MDA5 even in the presence of RLR agonists. In our hands, overexpression of tagged or untagged human DDX60 did not potentiate IFN induction alone or in response to viruses or RLR agonists. Moreover, unlike RIG‐I, DDX60 did not appear to capture stimulatory RNA as demonstrated by IP studies of epitope‐tagged DDX60 in infected or transfected cells. Finally, we found that cells or mice deficient in DDX60 displayed no alterations in IFN‐α/β responses to RNA viruses or to defined RIG‐I or MDA5 agonists. We also investigated a possible function of the helicase in other innate signaling pathways. DDX60 was not required in the cytokine response triggered by cytosolic DNA and cyclic dinucleotides, several TLR agonists, and the Dectin‐1 agonist, curdlan. Altogether, our data contrast with those of Miyashita et al. and Oshiumi et al. 21, 22. Whether the discrepancy between the studies can be attributed to slight differences in the KO cells generated, the use of different cell types, or other experimental details remains to be clarified and may be best resolved by a side‐by‐side analysis of the two DDX60 KO models. Nevertheless, our data argues that, in the mouse, DDX60 is dispensable for the induction of IFN‐α/β and other cytokines in response to a vast number of innate stimuli. We can obviously not rule out that DDX60 may have roles in induction of cytokines not measured here and in primary cell types yet to be tested.

We attempted to assess other possible functions for DDX60 in antiviral immunity. In vitro and in vivo infection studies in DDX60‐deficient cells and/or mice revealed no alteration in the susceptibility to multiple RNA viruses (IAV, SINV, and EMCV), DNA viruses (HSV‐1 and VACV), and one bacterium (*L. monocytogenes*). Thus, these data indicate that under our experimental conditions DDX60 does not function as a broadly acting infection restriction factor. Rather, we propose that DDX60 might act as an antiviral factor that is highly specific for a particular virus or class of viruses. This proposal is supported by the recent observation that ectopic expression of DDX60 in human cells is able to restrict the replication of HCV but not of yellow fever virus, West Nile virus, Chikungunya virus, Venezuelan equine encephalitis virus, or human immunodeficiency virus 1 23. Because of the tropism of the virus, the specific functions of DDX60 in restricting HCV replication cannot be investigated in mice, but it will be important to determine whether they are in any way related to the general properties of Ski2‐like helicases, such as promotion of RNA decay *via* the RNA exosome 21, 22, 49.

## Materials and methods

### Reagents

Poly(dA:dT) was purchased from Sigma‐Aldrich (St. Louis, MO), poly(I:C) from Amersham (Amersham, UK), and short (low molecular weight) poly(I:C) from InvivoGen (San Diego, CA). Cyclic diguanosine monophosphate (c‐di‐GMP, catalog number C‐057) and cyclic diadenosine monophosphate (c‐di‐AMP, catalog number C‐088) was purchased from BioLog (Hayward, CA). The *L. monocytogenes* strain 10403S coupled to ovalbumin (OVA) and erythromycin resistant was a kind a gift from Leo Lefrançois (University of Connecticut, USA). Recombinant murine granulocyte‐macrophage colony‐stimulating factor (GM‐CSF) was made by Cancer Research UK. Custom oligos used in this study were purchased from Sigma‐Aldrich. Recombinant IFN‐A/D was a gift from I. Kerr (Cancer Research UK). IVT‐RNA, Vero‐EMCV‐RNA, and IAV vRNA are as described in 33, 34.

### Plasmids and cloning

Both the pcDNA3.1‐3xFlag‐hRIG‐I and pcDNA3.1‐3xFlag‐hMDA5 were described before 32, 34. The control pcDNA3.1/V5‐His‐TOPO *lacZ* is from Invitrogen. The 3xFlag‐hMAVS was PCR‐amplified from cDNA from HEK293 cells using the following primers: fwd‐GCCGCCATGGACTACAAAGACCATGACGGTGATTATAAAGATCATGACATCGATTACAAGGATGACGATGACAAGCCGTTTGCTGAAGACAAGAC) and rev‐CTAGTGCAGACGCCGCCGGTAC and inserted into pcDNA3.1/V5‐His© TOPO® TA (Invitrogen). The following constructs: pcDNA3.1‐MYC‐hRIG‐I, peGFP.C1‐hTBK1, peGFP.C1‐h IRF‐3‐5D, and pcDNA3.1 hRIG‐I‐N aa(1‐229) have been described 30, 50.   The N‐terminal‐tagged eGFP, Flag, or 3xHA full‐length hDDX60 expression plasmids (EX‐H5198‐M29, EX‐H5198‐M11, and EX‐H5198‐M06, respectively) were purchased from GeneCopoeia (Rockville, MD). DDX60 plasmids were amplified from Flag‐DDX60 using specified primers before cloning into the pcDNA3.1/V5‐His© TOPO® TA vector from Invitrogen (Waltham, MA). The following primers were used: DDX60 (1‐1713aa) fwd‐ATGGAAAGAAATGTTCTTACAACATTTT, rev‐TTAGACTTTGTTTAACTTTTCCCAA; 3xFlag‐DDX60 (1‐1713aa) fwd‐GCCGCCATGGACTACAAAGACCATGACGGTGATTATAAAGATCATGACATCGATTACAAGGATGACGATGACAAGGAAAGAAATGTTCTTACAACATTTT, rev‐TTAGACTTTGTTTAACTTTTCCCAA; 3xFlag‐DDX60 (1‐771aa) fwd‐GCCGCCATGGACTACAAAGACCATGACGGTGATTATAAAGATCATGACATCGATTACAAGGATGACGATGACAAGGAAAGAAATGTTCTTACAACATTTT, rev‐AGAGCTCTCGCTGCCATGTGT; 3xFlag‐DDX60 (769‐1713aa) fwd‐GCCGCCATGGACTACAAAGACCATGACGGTGATTATAAAGATCATGACATCGATTACAAGGATGACGATGACAAGGAGCTCCTTGATGTTGTGGATAAGA, rev‐TTAGACTTTGTTTAACTTTTCCCAA; MYC‐DDX60 (751‐1713) fwd‐ATGGAGCAGAAACTCATCTCTGAAGAGGATCTGAGAAAAGACCCAGATCCCAG, rev‐TTAGACTTTGTTTAACTTTTCCCAA. DDX60L was cloned by PCR amplification of cDNA prepared from A549 lung epithelial cells treated overnight with IFN‐A/D (1000 IU/mL) using the following primers: fwd‐ATGGGGTCAAAGGATCATGCA and rev‐TTATTCTAAATGATTTTGACTCATTTGAATTTGC. Flag and MYC‐tagged constructs were cloned as detailed above into the pcDNA3.1/V5‐His© TOPO® TA vector using the following primers: 3xFlag‐DDX60L (1‐1707aa) fwd‐GCCGCCATGGACTACAAAGACCATGACGGTGATTATAAAGATCATGACATCGATTACAAGGATGACGATGACAAGGGGTCAAAGGATCATGCA, rev‐TTATTCTAAATGATTTTGACTCATTTGAATTTGC and MYC‐DDX60L (731‐1707aa) fwd‐ATGGAGCAGAAACTCATCTCTGAAGAGGATCTGAGAAAAGATCGGGATCCCAG and rev‐TTATTCTAAATGATTTTGACTCATTTGAATTTGC. The *Ddx60* 5′ arm probe was cloned from purified C57BL/6J genomic DNA into pcDNA3.1/V5‐His using the TOPO®TA Expression Kit (Invitrogen), using the following oligos: fwd‐GGGATAAAGGTTGTTACAGCAGG and rev‐GTTTACCTATGTGCCCACGAGC. The Neo probe was cloned using fwd‐GATTGAACAAGATGGATTGCACGC and rev‐AATATCACGGGTAGCCAACG oligos. All constructs were fully sequenced.

### Nucleic acid analysis

Real‐time quantitative (Q‐)PCR analysis was carried out as described using the following oligos: *ifnb1* (Mm00439546_s1; Applied Biosystems, Waltham, MA), *mDdx58* (*rig‐i*, Mm00554529_m1 (Applied Biosystems), *mDdx60* (Mm_Ddx60_2_SG QuantiTect Primer Assay,Qiagen, QT01074248), *mGapdh* (4352932E, Applied Biosystems), *hGAPDH* (402869, Applied Biosystems), *hDDX60* (fwd‐TCCCAAAGCTGATAAAGAAGCCC and rev‐CATGTATTAGGCTTTGATCCACATTTCTG), *hDDX60L* (fwd‐CCAGGAATAAAGACTCAACTTTGG and rev‐CCTCTTCTTCCAGCACGACC), and lacZ fwd‐ATGGAAGATCCCGTCGTTTTACAACG and rev‐CAACTGTTGGGAAGGGCGAT.

### Cells

NIH3T3, HEK293T, Vero were from Cancer Research UK and grown in D10 media (Dulbecco's Modified Eagle Medium [Gibco, Invitrogen] containing 10% fetal calf serum (FCS, Autogen Bioclear, Nottingham, UK; v/v), 100 U/mL penicillin, 100 U/mL streptomycin, 0.3μg/mL glutamine). HeLa cells, also from Cancer Research UK, were grown in M10 (minimum essential media [Gibco, Invitrogen] containing 10% FCS [v/v], 100 U/mL penicillin, 100 U/mL streptomycin, 0.3 μg/mL glutamine). HEK293 cells were a gift from Friedemann Weber (Freiburg, Germany) and grown in D10 as were MEFs. All cells used in this study were mycoplasma negative, as tested by Crick Cell Services core technology facility, and cultured on tissue culture treated polystyrene plates (Falcon, Waltham, MA) in an incubator with 5–10% CO_2_ and at 37°C. HEK293 cells stably expressing 3xFlag–hRIG‐I have been described 34. Flag‐hDDX60 stable clones were generated in a similar fashion using the Flag‐hDDX60 (EX‐H5198‐M11) plasmid from GeneCopoeia. HEK293 Flag‐hDDX60 stable clone F2‐E4 was used for experiments. GM‐CSF‐derived BMMCs were prepared as described 51. Primary *mavs^−/−^* and control MEFs were a kind gift from Jürg Tschopp (deceased) and were grown in D10 media. These cells were immortalized with simian virus 40 large T antigen as described 32. *Ddx60^−/−^*and littermate control MEFs were prepared from 12.5‐day embryos using standard protocols. Splenocytes were isolated as described 52.

### Mice

All mice used in this study were bred in specific pathogen‐free conditions by the The Francis Crick Institute Biological Resources Unit. Experiments were performed in accordance with national and institutional guidelines for animal care and were approved by the Animal Ethics Committee Review Board. WT C57BL/6Jax was purchased from Jackson Laboratories (Bar Harbor, ME). The C57BL/6 *mavs^−/−^* (also known as *cardif^−/−^)* mice were a kind gift from Jürg Tschopp (deceased). *Ifnar^−/−^* mice were a kind gift from Michel Aguet, University of Zürich (original strain name: B6.129S2‐Ifnar1*^tm1Agt^*), and backcrossed 14 times to C57BL/6J by the Immunobiology laboratory. Please see Supporting Information Material and Methods regarding the generation of DDX60 KO mice.

### In vivo mouse experiments

For infection studies, age/sex littermate‐controlled 8‐ to 10‐week‐old mice were used. Following pathogen administration, animals were monitored three times a day. Weights were also registered at the same time each day. If mice developed any serious clinical signs of disease or lost >20% of their weight, the humane endpoint was deemed to have been reached and the animals were killed.

To monitor murine IFN‐α following i.p. EMCV infection (1 × 10^6^ PFU in 100 μL of PBS [Dulbecco's phosphate‐buffered saline; Gibco, Invitrogen]) using a 27‐G needle and a 1 mL syringe, male mice were used. Blood was collected by cardiac puncture, placed in a 1.5 mL tube (Eppendorf, Hamburg, Germany) and left at 37°C for 10 min. Coagulated blood was spun out and serum was pipetted into a fresh tube. Samples were either used directly for enzyme‐linked immunosorbent assay (ELISA) or stored at −80°C. To monitor susceptibility to EMCV infections, male mice were infected in the peritoneal cavity with 200 PFU of virus diluted in 100 μL of PBS.

For *L. monocytogenes* infections, 8‐ to 10‐week‐old female mice were placed in a heated chamber for 5–10 min before being infected intravenously (i.v.) in the lateral tail vein with 1.6 × 10^5^ colony‐forming units (CFUs) in 100 μL of PBS using a 27‐G needle and a 1 mL syringe. Forty‐eight hours later, mice were sacrificed and organs harvested (spleen and liver). The organ's bacterial burden was assessed as detailed in 53. For HSV‐1 infections, 10‐ to 12‐week‐old male mice were treated as for *L. monocytogenes* infections and infected i.v. with 1 × 10^7^ PFU in 100 μL of PBS. For influenza infections, 10–12 weeks old female mice were anesthetized i.p. with 10 μL/g of body weight with a mix of 10 mg/mL of ketamine (Ketaset, Fort Dodge Laboratories, New York City, NY) and 0.5 mg/mL of xylosine (Xylacare®, Animalcare, Nether Poppleton, UK) in PBS. To protect their eyes, Lacri‐lube (Actavis, Parsippany, NJ) was applied. Mice were then infected by intranasal administration of 100 PFU (PR8 WT) or 200 PFU (Eng195) in 25 μL PBS. Liquid was carefully administered drop‐by‐drop into the left nostril using a 20 μL tip and pipette aid (Eppendorf). For SINV infections, male mice were injected subcutaneously in the ventral flank with 100 PFU of virus (in 100 μL of PBS).

### Virus strains

All infection work was carried out according to the requirements for handling biological agents in Advisory Committee on Dangerous Pathogens (ADCPs) hazard groups. The following viral strains were used: Sev Cantell Strain (ATCC VR‐907); IAV strain A/Puerto Rico/8/1934 H1N1 Cambridge variant WT (PR8 WT) and delta NS1 gene (PR8ΔNS1; kind gifts from T. Muster (University of Vienna, Austria); IAV pandemic H1N1 strain A/England/195/09 (a kind gift from W. Barclay [Imperial College London, UK]), EMCV and SINV (kind gifts from Ian Kerr); HSV‐1 (ATCC VR‐1493); reovirus T3D (a kind gift from T. Dermody, Vanderbilt University, TN); as well as MVA, WR‐A3L‐RFP, and MVA‐A3L‐RFP (kind gifts from Michael Way [The Francis Crick Institute, London, UK]).

### Infection of MEFs and BMMCs

All infections were carried out by first washing the cells in FCS‐free media, adding virus suspension diluted in FCS‐free media, leaving the cells for 1 h at 37°C/5%CO_2_, and adding an equivalent volume of media containing 20% FCS (v/v; unless indicated). For Figure 5 and Supporting Information Figure 4, 2 × 10^5^ *Ddx60^+/+^, Ddx60^+/−^*, or *Ddx60^−/−^* BMMCs were plated per well in a 24‐well plate in 500 μL of media. Twenty‐four hours later, the supernatant was removed, and 200 μL of virus suspension was added. For Figure 8A and Supporting Information Figure 6B, 2 × 10^5^ *Ddx60^+/+^*, *Ddx60^+/−^*, or *Ddx60^−/−^* primary MEFs were plated in the evening in a 6‐well plate in 2 mL of media. The next morning, cells were washed once in FCS‐free media and 1 mL of virus suspension (PR8 WT, WR‐A3L‐RFP, or MVA‐A3L‐RFP) was added for 1 h. After this time, media was removed and 2 mL of fresh D10 was added.

### IP of Flag‐RIG‐I or Flag‐DDX60 with nucleic acids

Eighty percent confluent 145 cm^2^ plates of HEK293 cells stably expressing Flag‐hRIG‐I or Flag‐hDDX60 were either transfected with 5 μg (0.1 μg/mL) of IVT‐RNA or 20 μg (1 μg/mL) of poly(dA:dT) using Lipofectamine 2000 (Invitrogen) or infected with SeV. Sixteen hours later, cells were washed with ice‐cold PBS and lysed in ice‐cold 0.5% NP‐40 lysis buffer A or B (buffer A: 0.5% NP40 (v/v), 20 mM Tris‐HCl, pH 7.5, 150 mM NaCl; 2.5 mM MgCl_2_; complete protease inhibitor [Roche Applied Sciences, Basel, Switzerland], 0.1 U/mL RNasin [Promega, Fitchburg, WI]; buffer B: 0.5% NP40 (v/v), 20 mM Tris‐HCl, pH 7.5, 100 mM NaCl; 1 mM EDTA; complete protease inhibitor, 0.1 U/mL RNasin). Lysates were left on ice for 30 min and centrifuged at 20 000 × *g* for 15 min to remove cell debris. Resulting supernatants were equally split and incubated with α‐Flag M2 antibody (Sigma‐Aldrich) or control mIgG1 (BD Biosciences, Franklin Lakes, NJ) for 1.5–2 h at 4°C on a rotating wheel before adding prewashed Gamma Bind Plus Sepharose beads (GE Healthcare, Little Chalfont, UK). Two hours later, beads were spun down and washed five times for 5 min with 0.5% NP‐40 lysis buffer. Beads samples were then split for RNA extraction or Western blotting. For Figure 7G, HEK293T cells were transfected with pcDNA3.1‐3xFlag‐hDDX60 or pcDNA3.1‐3xFlag‐hRIG‐I. Forty‐eight hours later, cells were lysed in 0.5% NP‐40 buffer A and Flag‐tagged proteins were immunoprecipitated as detailed above. Washed beads were then incubated with 3.5 μg of IAV vRNA for 1.5 h at 4°C on a rotating wheel. Beads were then washed five times for 5 min with 0.5% NP‐40 lysis buffer A before RNA was extracted.

### Co‐IP studies

For Figure 6A, 1×10^6^ HEK293T cells were transfected using Lipofectamine 2000 with 1 μg each of eGFP‐hDDX60 (EX‐H5198‐M29) plasmid and 0.3 μg of pcDNA3.1‐3xFlag‐tagged hDDX60, hMDA5, hRIG‐I, or hMAVS. Twenty‐four hours later, cells were washed in PBS and lysed on ice in 250 μL of cold 0.5 % NP40 buffer A. The lysate was centrifuged at 20 000 g for 10 min and the supernatant was collected. After keeping input material, the supernatant was incubated with α‐Flag M2 antibody on a rotating wheel at 4°C. One and a half hour later, 25 μL of Gamma Bind Plus Sepharose beads (GE Healthcare) was added and rotated for 2 h. Beads were washed five times in lysis buffer before the GFP‐hDDX60 containing complexes were recovered by boiling the beads in Laemmli buffer (10% glycerol [v/v], 5% β‐mercaptoethanol [v/v], 3% SDS [w/v], bromophenol blue, 0.5× Upper Tris buffer) for Western blot analysis 34. Membranes were probed with α‐Flag‐M2‐HRP (Sigma‐Aldrich; 1:10 000), α‐GFP‐3E1 (Crick Antibody Service; 1:1000), or α‐GFP‐HRP (Miltenyi Biotec, Bergisch Gladbach, Germany; 1:5000) antibodies. For Figure 6B and D, HEK293 cells stably expressing Flag hDDX60 or 3xFlag‐hRIG‐I were used and transfected with pcDNA3.1‐MYC‐hRIG‐I or treated with different RLR stimuli as indicated. The α‐Flag‐M2 and Gamma Bind Plus Sepharose beads were used for IP as detailed above. For Figure 6D, mIgG1 was included as control. Following Western blot analysis, membranes were probed with α‐Flag‐M2‐HRP, α‐MYC (Sigma‐Aldrich; 1:5000), or α‐DDX60 (0.1‐0.2 μg/mL, see Supporting Information Materials and methods for details on the generation of the α‐DDX60 antibodies). For Figure 6C, one million HEK293T cells were transfected using Lipofectamine 2000 with 1 μg each of 3xHA‐hDDX60 (EX‐H5198‐M06) plasmid and 1 μg of pcDNA3.1‐3xFlag‐tagged hDDX60, or hRIG‐I. Forty‐eight hours later, cells were washed in PBS and lysed on ice in 750 μL of cold 0.5 % NP40 buffer A. IP was carried out as detailed above but using 10 μL of α‐HA‐3F10 affinity matrix (Roche Applied Science). Western blots were probed with α‐Flag‐M2‐HRP or α‐HA‐3F10‐HRP (Roche Applied Science; 1:3000) antibodies. When required, secondary α‐mouse‐HRP or α‐rabbit‐HRP antibodies were used (Southern Biotech, Birmingham, AL; 1:5000).

### Detection of IFN stimulatory activity

For assays using MEFs and BMMCs, 1–5×10^4^ cells/mL were plated into wells of 24‐ or 48‐well plates and transfected with test or control RNAs/DNA using Lipofectamine 2000. After 16–24 h incubation, murine IFN‐α (multiple subtypes) in culture supernatants was quantified by ELISA as described 54 or RNA was extracted. Serum levels of murine IL‐6 were measured by cytometric bead array (BD Pharmingen) and mIFN‐β levels were determined by ELISA (PBL Assay Science, Piscataway, NJ). The luciferase promoter reporter assays were employed as described 33. Firefly luciferase activity was normalized to Renilla luciferase, and fold inductions were calculated relative to a control transfection using water only (F‐luc/R‐luc).

### Flow cytometry

For the intracellular staining of the influenza protein NS1 in Figure 8A, cells were harvested, washed twice in PBS to remove any traces of FCS, and fixed in 4% paraformaldehyde (from Electron Microscopy Services diluted in PBS, v/v) for 15 min at room temperature. After washing cells twice more, they were resuspended in fluorescence‐activated cell sorting (FACS) buffer (PBS with 3% FCS [v/v] and 0.02% NaN_3_ [w/v]) for blocking and left overnight at 4°C. Cells were pelleted and resuspended in 100 μL of Cy3 labeled anti‐NS1‐1A7 antibody (gift from Jon Yewdell [NIH]), final 10 μg/mL diluted in Fix & Perm Permeabilization medium—Buffer B from An Der Grub (Caltag, Buckingham, UK). After 1 h incubation on ice, cells were washed in FACS buffer before analyzing samples by FACS. For gB‐pentamer staining, 300–400 μL of blood was collected into a heparin tube from mice 8 days after HSV‐1 infection. Following 3 min of red blood cell lysis (Sigma), cells were pelleted at 1700 rpm for 5 min (Beckman, Allegra6 Centrifuge) and washed twice in FACS buffer. Cells were then stained with H‐2Kb‐SSIEFARL pentamers (ProImmune, Oxford, UK) to detect gB‐specific CD8^+^ T cells for 45 min at room temperature. Anti‐FcγRIII/II (BD Biosciences) was added to block nonantigen‐specific binding of immunoglobulins. After washing cells once in FACS buffer, activated CD8^+^ T‐cell population was stained with anti‐CD8‐FITC and anti‐CD44‐allophycocyanin (BD Biosciences) for 45 min at room temperature. Cells were washed twice in FACS buffer before analysis. All data were acquired on FACSCalibur (BD Biosciences).

### β‐Galactosidase activity assay

Chlorophenolred‐β‐d‐galactopyranoside (CPRG) solution was freshly reconstituted from a 10× stock (30 mM chlorophenolred‐β‐d‐galactopyranoside [CPRG from Roche Applied Sciences] in Z buffer [0.6 M Na_2_HPO_4_.7H_2_O, 0.4 M Na_2_HPO_4_.H_2_O, 0.1 M KCl, 0.01 M MgSO_4_.7H_2_O, 0.5 M β‐mercaptoethanol]) and 0.1 mL of 1× solution (1× CPRG, 0.1% NP‐40 [v/v], 10 mM MgCl_2_ in PBS) was added to 1 × 10^6^ MEFs or 1 × 10^6^ total splenocytes in 0.1 mL of PBS in a 96‐well plate. The reaction was allowed to take place at 37°C for 1–4 h and chlorophenol red was measurable by spectrophotometry (A595–A655).

### Cell proliferation and cell viability assays

Cell proliferation assays were performed using Cell Titer 96® kit from Promega according to instructions from the supplier. Cell viability was determined by Trypan blue exclusion assay using the Vi‐Cell^TM^ XR Cell Viability Analyzer (Beckman Coulter, Brea, CA).

### Microscopy analysis

HeLa cells were transfected with eGFP‐hDDX60 for 48 h before being harvested and 200 000 cells were allowed to adhere in phenol‐free media (Gibco, Invitrogen) for 15 min at 37°C to MatTek dishes (MatTek Corporation, Ashland, MA) coated with 10 μg/mL fibronectin (Sigma‐Aldrich). Images were captured with Zeiss LSM 510 confocal microscope (Oberkochen, Germany) and analyzed using LSM 510 (Zeiss) and ImageJ (NIH) softwares.

### Protein analysis

For Figure 1A, human protein sequences were extracted from RefSeq database [http://www.ncbi.nlm.nih.gov/RefSeq] and aligned with ClustalW using default parameters. The BLOSUM scores were calculated from separate pairwise alignments, which were then rescored to give percent identity score. INTERPRO [release 26.0 http://www.ebi.ac.uk/interpro] was used to scan human protein sequences for the presence of functional domain/signature recognition models using the InterProScan facility. The Hel1 domain containing the Walker B motif in each helicase was identified by searching SMART database with the DEAD (asp‐glu‐ala‐asp)‐like helicase model accession number SM00487 or Interpro ID: IPR014001. Dendrograms showing sequence similarities were generated with the neighbor‐joining method as implemented by the ClustalX multiple sequence alignment program. The resulting dendrogram was visualized with the Dendroscope interactive viewer 55.

### Promoter analysis

Three kilobases of upstream sequence from the start codon was extracted for human DDX60 (NM_017631.5), human DDX60L (NM_001012967.1) from hg19 assembly and mouse DDX60 (NM_001081215.1) from mm9 assembly using the USCS browser (http://genome.ucsc.edu) 56. A consensus sequence for IFN‐stimulated response element (ISRE)—GAAA[GCT][CGT][GA]AAA—was built using well‐characterized IFN responsive genes. The “fuzznuc” program within EMBOSS was used to determine the location of the ISRE motif in both the sense and antisense strand 57.

### Computer programs used and statistical analysis

Microsoft Office® for Mac (version14.4.1), Adobe Photoshop® and Illustrator® CS5.1 (version 12.1×64), Adobe Acrobat X Pro (version 10.0.0), and Papers2® (version 2.1.17) softwares were used to prepare this document. FACS data were registered on CellQuest and analyzed using FlowJo® (version 9.4.7). Statistical analysis and the majority of graphs in this study were computed using the GraphPad PRISM® program (version 6.0d).

## Conflict of interest

The authors declare no commercial or financial conflict of interest.

**Abbreviations**
BMMC: BM‐derived myeloid cells
cGAS: cyclic GMP‐AMP synthase
EMCV: encephalomyocarditis virus
IAV: influenza A virus
IRF‐3: IFN regulatory factor 3
ISG: IFN‐stimulated gene
IVT‐RNA: in vitro transcribed RNA
LGP2: laboratory of genetics and physiology 2
MAVS: mitochondrial antiviral‐signaling protein
MDA5: melanoma differentiation‐associated gene 5
MVA: modified vaccinia Ankara
poly(dA:dT): poly(deoxyadenylic‐deoxythymidylic) acid sodium salt
RFP: red fluorescent protein
RIG‐I: retinoic acid inducible gene 1
RLR: RIG‐I‐like helicase
SeV: Sendai virus
SINV: Sindbis virus
STING: stimulator of IFN genes
VACV: vaccinia virus

## Supporting information

As a service to our authors and readers, this journal provides supporting information supplied by the authors. Such materials are peer reviewed and may be re‐organized for online delivery, but are not copy‐edited or typeset. Technical support issues arising from supporting information (other than missing files) should be addressed to the authors.

Supplementary Material
