# Supplementary material for: Mouse superkiller‐2‐like helicase DDX60 is dispensable for type I IFN induction and immunity to multiple viruses
Source: Eur J Immunol. 2015 Oct 12;45(12):3386–403. doi: 10.1002/eji.201545794 (PMC4833184; doi:10.1002/eji.201545794)
Supplement: Supplementary file 1 — Supplementary Material [file EJI-45-3386-s001.zip › eji3448-sup-0001-text.pdf]

European Journal of Immunology

Supporting Information
for
DOI 10.1002/eji.201545794

Delphine Goubau, Annemarthe G. van der Veen, Probir Chakravarty, Rongtuan Lin, Neil Rogers, Jan Rehwinkel, Safia Deddouche, Ian Rosewell, John Hiscott and Caetano Reis e Sousa-C

Mouse superkiller-2-like helicase DDX60 is dispensable for type I IFN induction and immunity to multiple viruses

© 2015 The Authors. *European Journal of Immunology* published by WILEY-VCH Verlag GmbH & Co. KGaA, Weinheim.

SUPPORTING INFORMATION

SUPPORTING FIGURES AND FIGURE LEGENDS

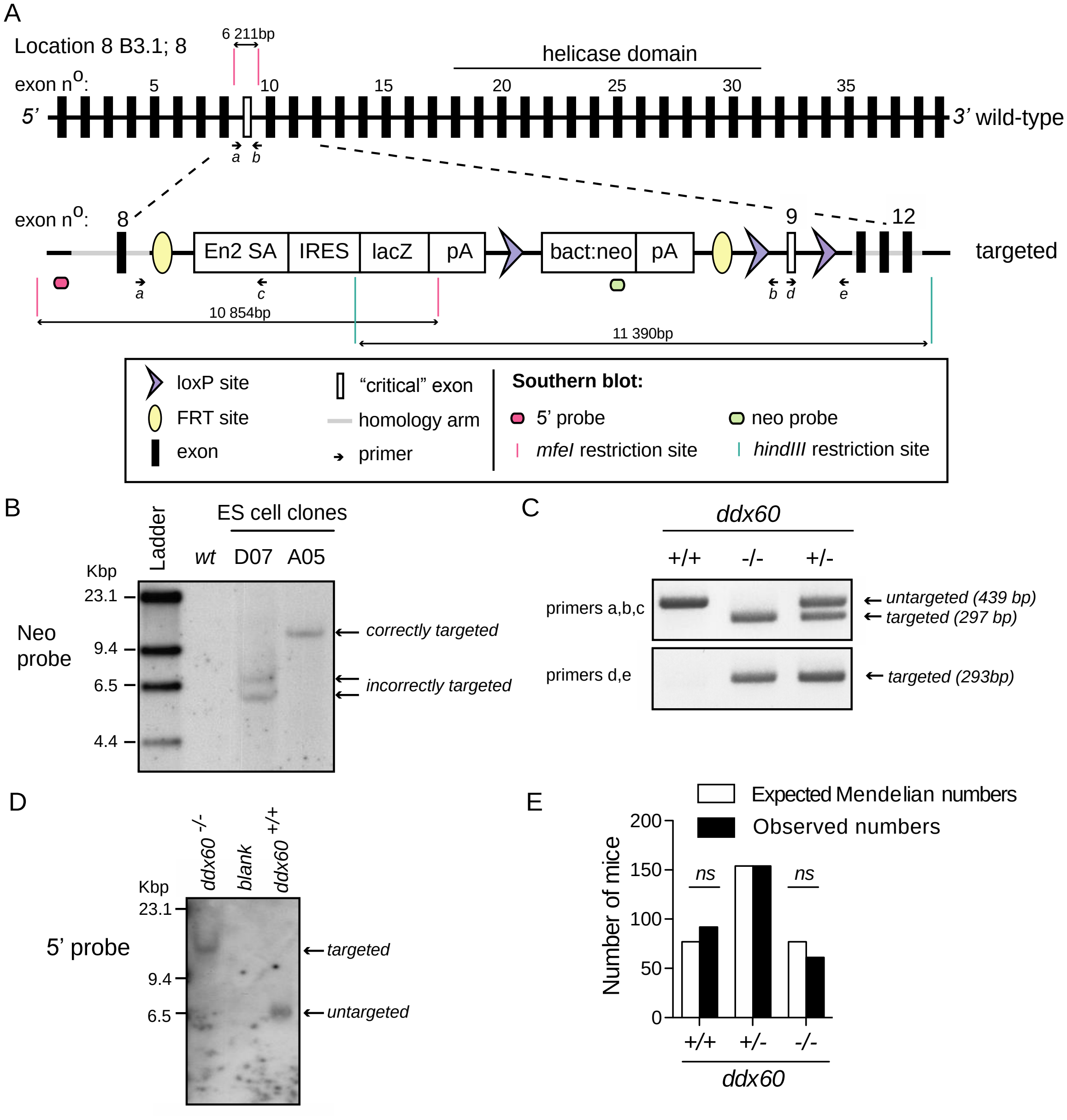

Supportive Figure S1 - Generation of ddx60 "knockout-first" mice.

(A) Schematic representation of the *ddx60*-targeting construct. C57BL/6 embryonic stem (ES) cells targeted with lacZ reporter-tagged, "knockout first"

conditional alleles were purchased from EUCOMM. Figure also details the wild-type *ddx60* allele as well as the primers, Southern blot probes and restriction sites used for genotype validations experiments conducted in (B-E). (B) DNA was extracted from *ddx60*-targeted ES cell clones A05 and B07 and analysed by Southern blot using the neomycin (neo) specific probe. (C) DNA was extracted from earsnips of mice from *ddx60* crosses and subjected to PCR analysis with indicated primers and products were analysed by gel electrophoresis. Data are representative of genotyped samples from $ddx60^{+/+}$, $ddx60^{+/-}$ and $ddx60^{-/-}$ mice. (D) Southern blot analysis of DNA purified from $ddx60^{+/+}$ and $ddx60^{-/-}$ mice probed with a 5'-end probe. (E) Graph shows the number of $ddx60^{+/+}$, $ddx60^{+/-}$ and $ddx60^{-/-}$ mice obtained from heterozygous crosses. Expected Mendelian numbers are also included for reference.

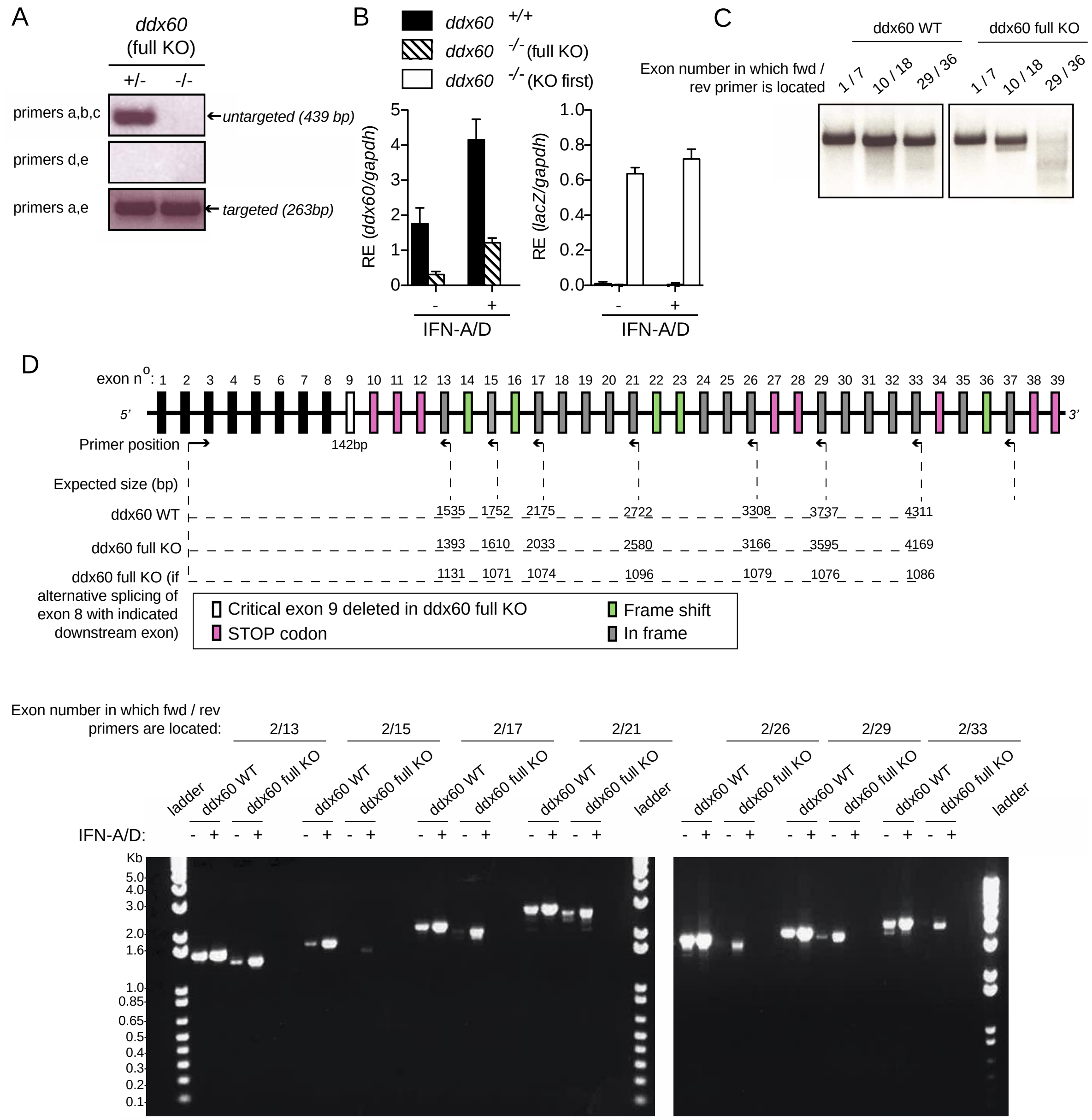

Supportive Figure S2 – Generation and validation of the ‘full” ddx60 knockout mice

To generate the “full” ddx60 knockout mice where the targeting cassette and exon 9 are deleted, the *Ddx60* “knockout first” mice (Supportive Fig. S1A) were first crossed with mice expressing flippase (hACTB-FLPe) to remove the targeting cassette within FRT sites and then with PGK-cre expressing mice to excise the

floxed exon 9. (A) DNA was extracted from earsnips of mice in ddx60 “full knockout” crosses and subjected to PCR analysis with the indicated primers (see Supportive Fig. S1A for primer details). PCR products were analysed by gel electrophoresis and confirm the removal of exon 9 in ddx60 “full knockout” mice. Data are representative of genotyped samples from “full” *ddx60*$^{-/-}$ and *ddx60*$^{-/-}$ knockout mice. (B) BMMCs were prepared from *Ddx60*$^{+/+}$, *ddx60*$^{-/-}$ (KO first) and *ddx60*$^{-/-}$ (full KO) mice were treated (+) or not (-) with recombinant IFN-A/D (16 h, 1000 IU/ml) and the relative expression of *ddx60* and *I*□□□ mRNA was assayed by RT-qPCR. Data were normalized to *gapdh*. A decreased but still significant level of *ddx60* transcripts in cells from *ddx60* full knockout compared to wild-type mice was observed. Additional analysis in (C) showed that whereas PCR products from exons 1 to 7 and 10 to 18 are amplifiable from cDNA prepared from wild-type or *ddx60* full knockout BMMCs pre-treated for 16 h with IFN-A/D, they are only detectable with wild-type cDNA when oligos amplifying exons 29 to 36 are used, indicating that incorrect splicing events between exon 8 and downstream exons has occurred. Yet, as deletion of exon 9 induces a frameshift mutation, these mutant transcripts are likely to be degraded by nonsense-mediated RNA decay. To further validate the ddx60 full KO mice, we investigated whether alternative splicing events occur in ddx60 full KO cells that could result in the expression of a truncated DDX60 ORF (D). After identifying the possible splicing events between exon 8 and downstream exons in ddx60 full KO that did not result in a frame shift or a stop codon, we performed PCR analysis using a forward primer spanning exons 2 and 3 and a series of reverse primers located in exons that could give a

truncated DDX60. cDNA from BMMCs treated of not with IFN-A/D (to increase DDX60 mRNA expression levels) from ddx60 full KO mice was used a template. Other than the PCR products resulting from the splicing of exon 8 to exon 10 in the ddx60 full KO, we did not observe any bands corresponding to the size expected from a truncated ORFs. Similarly, only one main PCR product corresponding to that of the expected ORF was detected when cDNA from DDX60 WT cells was used as template.

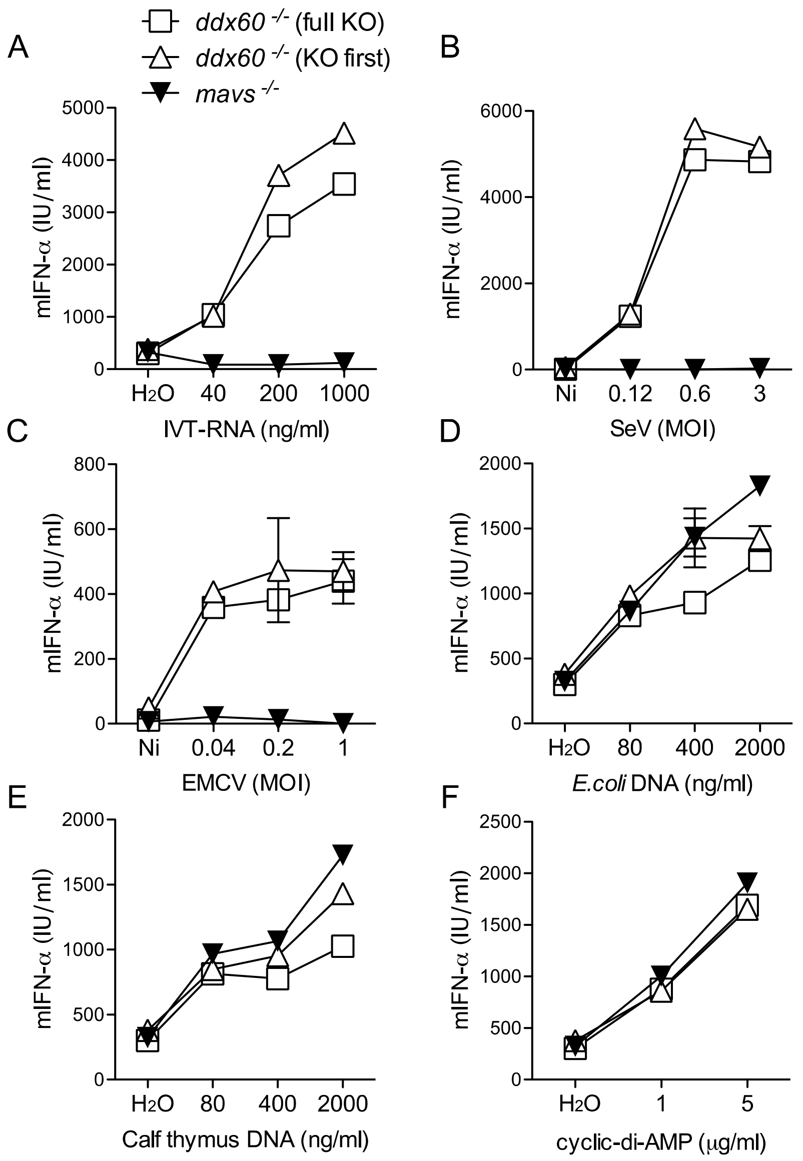

Supportive Figure S3 - Ddx60 “knockout first” and “full knockout” mice respond similarly to innate stimuli.

BMMCs were prepared from *ddx60*$^{-/-}$ (KO first), *ddx60*$^{-/-}$ (full KO) and *mavs*$^{-/-}$ mice. Cells were either transfected as indicated with different concentrations of IVT-RNA (A), *E.coli* DNA (D), calf-thymus DNA (E), or cyclic-di-AMP (F), or infected with different MOIs of SeV (B) or EMCV (C) for 24 h. mIFN-α protein levels were

assessed in culture supernatants by ELISA. Non-infected (Ni) and water only controls were also included. The mean (±s.d.) of triplicate technical replicates is shown. For all experiments one representative of three independent experimental repeats is shown.

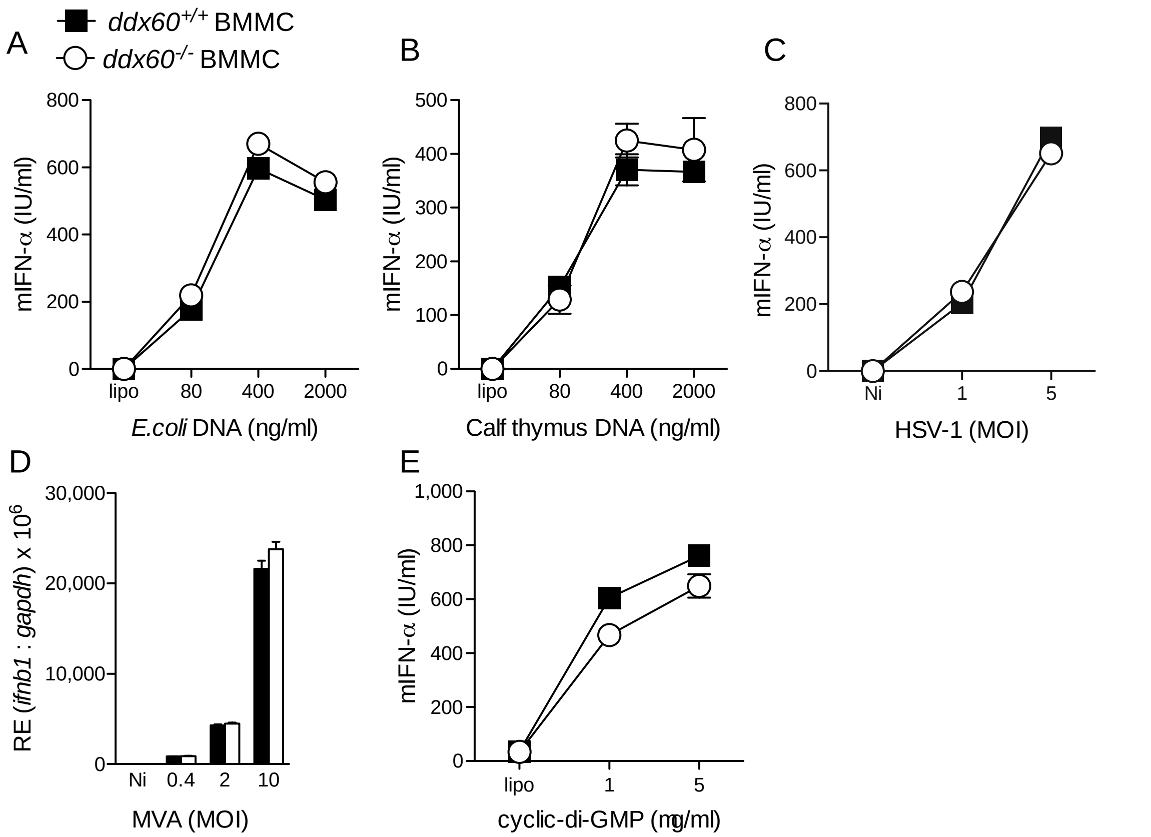

Supportive Figure S4 - Production of IFN-α/β is not affected in *ddx60*$^{-/-}$ BMMCs responding to stimulation with agonists of the cytosolic DNA sensing pathway. *Ddx60*$^{+/+}$ and *ddx60*$^{-/-}$ BMMCs were transfected with different concentrations of (A) *E.coli* DNA, (B) calf thymus DNA, or (E) cyclic-di-GMP. Water was used as a transfection control. In (C and D) cells were infected or not (Ni) with HSV-1 or MVA at indicated MOIs. For (A-C, and E) levels of mIFN-α in 24 h cell culture supernatants were assessed by ELISA. For (D), cells were harvested 6 h post-infection and the relative expression (RE) of murine *ifnb1* was assessed by quantitative PCR. Data were normalized to *gapdh.* The mean (±s.d.) of triplicate

technical replicates is shown. For all experiments one representative of three independent experimental repeats is shown.

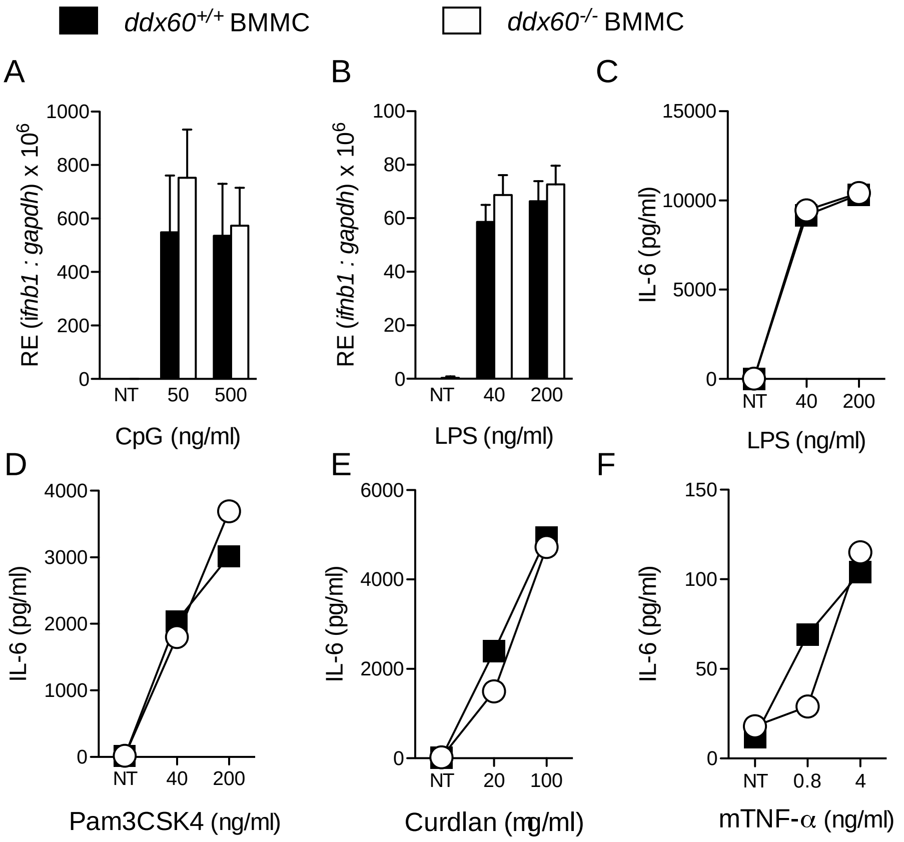

Supportive Figure S5 - Treatment of *ddx60*-sufficient or deficient cells with different TLR-stimuli, curdlan and TNFα induces similar levels of cytokines. *Ddx60*$^{+/+}$ and *ddx60*$^{-/-}$ BMMCs were treated with indicated concentrations of (A) CpG DNA, (B, C) LPS, (D) Pam3CSK4, (E) curdlan, or (F) recombinant murine TNF-α (mTNF-α). For (A) and (B), cells were harvested 6 h post-treatment, RNA extracted and the RE of murine *ifnb1* was assessed by quantitative PCR. Data were normalized to *gapdh*. The mean (±s.d.) of triplicate technical replicates is shown. For (C-F), cell culture supernatants were collected 24 h post-treatment and levels of murine IL-6 were assessed by CBA. Non-treated (NT) cells were also

included. For all experiments one representative of three independent experimental repeats is shown.

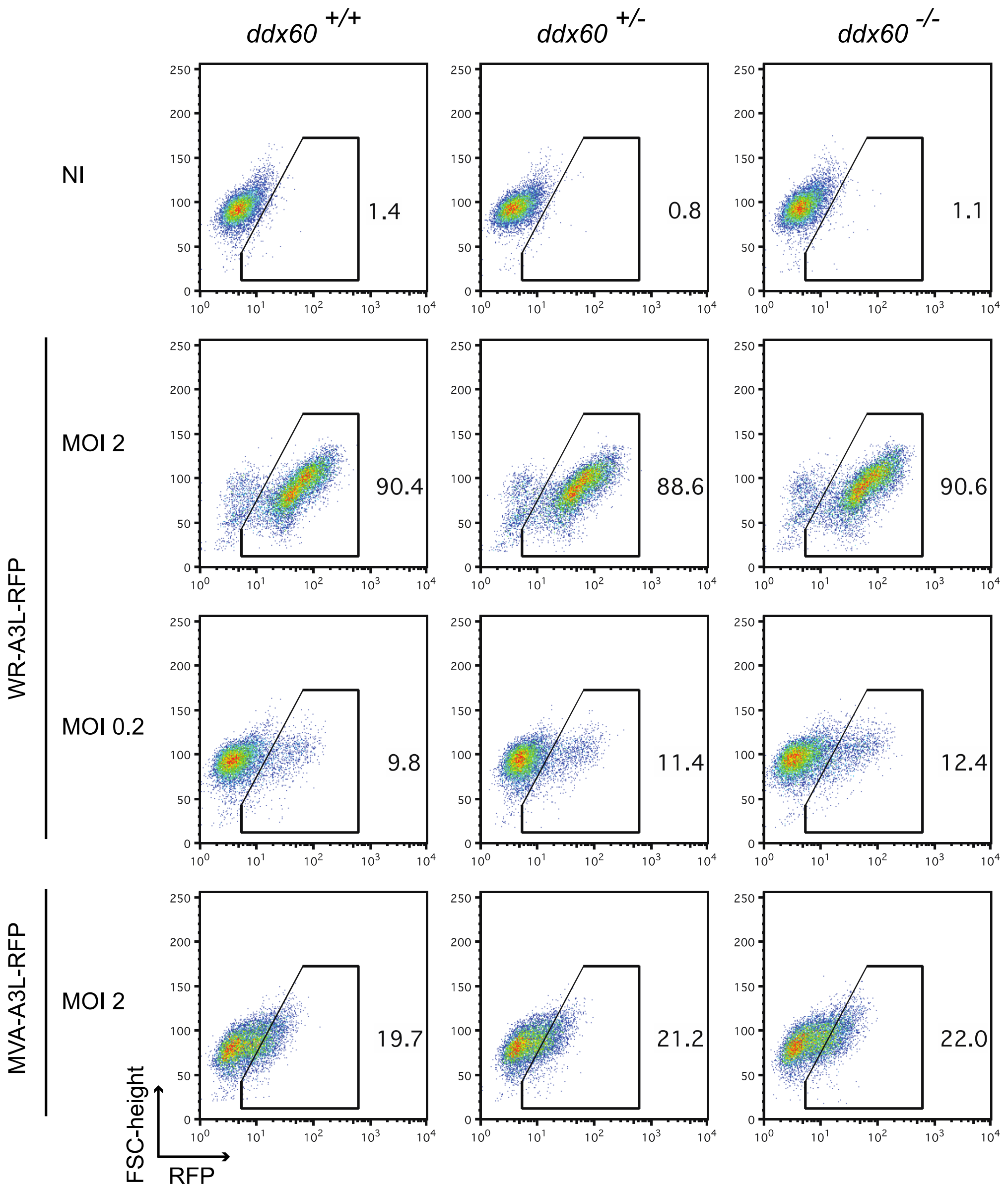

Supportive Figure S6 - Expression of ddx60 does not restrict vaccinia virus infection in MEFs.

MEFs of indicate genotypes were infected with vaccinia viruses WR-A3L-RFP or MVA-A3L-RFP at indicated MOIs for 18 h. Cells were then harvested, fixed and analysed by FACS for A3L-RFP expression. Non-infected (Ni) cells were also

included. Numbers represent the percentage of cells within the indicated gate. One representative of two independent experimental repeats is shown.

MATERIALS AND METHODS FOR SUPPORTING INFORMATION

Reagents

Ultra-pure calf-thymus DNA (catalog number D4764) was purchased from Sigma-Aldrich. CpG DNA (catalog number tlrl-1668) and Pam3CSK4 (catalog number tlrl-pm2s) are from Invivogen (San Diego, CA). *E.coli* DNA (catalog number 14380) was from USB (Cleveland, OH). *Salmonella abortus* derived LPS (catalog number ALX-581-009-L002) was from Alexis Biochemicals (Farmingdale, NY) and curdlan (catalog number 034-09901) from Wako (Osaka, Japan). The recombinant murine TNFα was from R&D (Minneapolis, MN).

Generation of anti-DDX60 antibody

Two human DDX60 peptides 753-765C (KDPDPRVQDFIPDC) and 812-823C (VYVAPTKALVNQC) were generated by CRUK-London Research Institute Peptide Synthesis Core Facility. These peptides were sent to Pettingill Technology Ltd. (Oxford, UK) where they were co-injected into rabbits to generate a polyclonal anti-DDX60 antibody. Final bleeds were then purified using Sulfo Link Immobilization kit for peptides (Pierce - Thermo Scientific, Waltham, MA) and the antibody was first eluted with 100mM glycine pH 2.5 followed by 100mM triethlyamine (TEA) pH 11.5. Elutions were then dialyzed (3500 MWCO, Pierce - Thermo Scientific), aliquoted and stored at -80°C. The antibody was validated by Western blot analysis using lysates of cells overexpressing tagged-versions of human DDX60 or of cells stimulated with IFN-A/D.

Southern blot

The Southern blot protocol used was adapted from the one obtained from Dr Ken Murphy's laboratory (St-Louis, MO). PCR labelling of the *ddx60* 5'-arm and neomycin probes was performed using the pcDNA3.1.ddx60 5'arm plasmid as a template and the PCR DIG (digoxigenin) Probe Synthesis kit (Roche Applied Sciences cat n° 11636090910) according to manufacturer's instructions. DIG-labelling efficiency was confirmed by gel electrophoresis. Genomic DNA was extracted from mice tail snips using phenol:chloroform:isoamylalcohol. Twenty micrograms of DNA was restriction-digested overnight at 37°C with either *hind III* for neomyocin probing or *mfeI* for 5' arm probing as directed by the supplier. Digested DNA was purified using phenol:chloroform:isoamylalcohol and resuspended in water and DNA loading dye (Qiagen, Limburg, Netherlands). Samples were loaded on a 0.8% agarose (w/v) TAE (40mM Tris base, 20mM acetic acid, and 1mM EDTA) gel along with 5μl of DIG ladder (Roche Applied Sciences, cat n° 11 218 590 910) and run at 60V. The gel was first stained with ethidium bromide, washed and imaged under UV transilluminator to verify the quality of the restriction digest. Following this, it was incubated in hydrolysis buffer (0.25M HCl) for 10 min, denaturing buffer (1.5M NaOH, 0.5M NaCl) for 30 min and neutralization buffer (1.5M NaCl, 0.5M Tris-HCl (pH 7.2), 10mM ethylenediaminetetraacetic acid (EDTA)) for 30 min. Buffer incubations were done with gentle rocking and preceded with briefly rinsing the gel in water. After overnight transfer onto a Hybond+ nylon membrane (Amersham Biosciences) by capillary transfer (20x saline-sodium citrate (SSC) made of 3M sodium chloride, 300mM trisodium citrate (pH 7.0 with HCl)), the DNA was UV-crosslinked onto the membrane

using UV-X-linker (Amersham Biosciences Hoefer UVC 500) set at 1200mJoules x 100. The membrane was then rinsed in water and pre-hybridized with the DIG Easy Hyb solution from Roche Applied Science for 30 min at 42°C on a rotating wheel. The DIG-labelled probe was denatured for 5 min at 95°C followed by rapid cooling on ice before being diluted in DIG Easy Hyb solution (2μl of probe / 1ml of solution). Following overnight hybridization at 42°C, the membrane was washed twice with wash buffer I (2x SSC, 0.1% SDS (w/v)) for 5 min at room temperature, twice with wash buffer II (0.2% SSC (v/v), 0.1% SDS (w/v)) for 15 min at 68°C, and once with wash buffer III (0.1M Maleic acid, 0.15M NaCl; pH 7.5, 0.3% Tween (v/v)), for 2 min. The membrane was subjected to immunological detection using anti-digoxigenin antibody conjugated to alkaline phosphatase following the procedure in the DIG nucleic acid detection kit from Roche Applied Science. The chemiluminescent substrate for alkaline phosphatase, CSPD, was then used for detection following the manufacturer's instructions (Roche Applied Sciences).

DDX60 knockout mouse generation

To generate the ddx60 knockout mice, two targeted ddx60 embryonic stem (ES) cell clones (A05 and D07) with conditional potential were purchased from European Conditional Mouse Mutagenesis Program (EUCOMM; Wellcome Trust Sanger Institute, project ID: 41373) a member of the International Knockout Mouse Consortium. The Cancer Research UK Transgenic Core Facility used the A05 ES clone for blastocyst injection and the resulting chimeras were crossed to wild-type C57BL/6Jax mice for transmission. To generate the ddx60 "full knockout" line, *ddx60*-targeted mice were

crossed to hACTB-FLPe C57BL/6J (strain name B6.Cg-Tg(ACTFLPe)9205Dym) mice, which express *FLP1* recombinase gene under the direction of the human β-actin (ACTB) promoter and PGK-Cre C57BL/6J mice (strain name B6.Cg-Tg(Pgk-cre)1Lni) mice, which directs the expression of Cre recombinase under the control of a phosphoglycerate kinase (PGK) promoter mice. Both the hACTB-FLPe C57BL/6J mice and the PGK-Cre C57BL/6J mice were obtained from the CRUK Biological Resources.

DDX60 knockout mouse genotyping

For the genotyping of *ddx60*$^{+/+}$, *ddx60*$^{+/-}$, and *ddx60*$^{-/-}$ mice, ear-snip DNA was used and PCR reactions carried out using GoTaq Flexi polymerase (Promega) using the following primers: primer “a” (ATATCGATGCACAAACTTACATTGGG), primer “b” (AGGAAAGGCAAATGGAAGGACTGC), primer “c” (CAACGGGTTCTTCTGTTAGTCC), primer “d” (TTAAGTGATGAGCCTTTGTTGAGG), and primer “e” (TGAACTGATGGCGAGCTCAGACC). For *ddx60* oligo combinations a/b/c, d/e or a/e, the following amplification program was used: 95°C for 30 sec, 62°C for 30 sec and 72°C for 30 sec, for 35 cycles. PCR products were separated on 0.8-2.0% agarose (w/v) gel prepared using TAE and pre-stained with ethidium bromide (Sigma). Bands were visualized using a UV transilluminator and a Dimage Xt digital camera (Minolta) mounted on a stand.

DDX60 full-knockout cells PCR validation

For Supportive Figure 2C, the following amplification program: 95°C for 30 sec, 62°C for 1 min and 72°C for 30 sec, for 35 cycles and the following primers exon 1 fwd:

ACTGTGTGCTTGCTTGTTAC; exon 7 rev: CCAAGAAGAGAGAACATGAGGTG; exon 10 fwd: CCAATCAAAGAAAGTTCCCAGG; exon 18 rev: ATAGTTATCCACCACATCCAGGAG; exon 29 fwd: GGTCGAAATTCTCTTCAGGAAAGG; exon 36 rev: TTCCAGACAGACAAACAAAGGGTG were used.

For Supporting Figure 2D, the following amplification program: 95°C for 30 sec, 62°C for 1 min and 72°C for 4.5 min, for 35 cycles and the following primers: exon 2 fwd: GCAGCAAGAATGGAAAAAAAATACACAGGT; exon 13 rev: CTGAGAGGTCTGTGGGAGTGC; exon 15 rev: AATTATTTCAGCCTTGGTCTC; exon 17 rev: CATTTTTATGCCATCTGATGCATC; exon 21 rev: GATGCTCCCAGATTTCCGCTCCAA; exon 26 rev: GGGCTCAGTTTCTTCAGCACCATC; exon 29 rev: ACCCGGTGAAACACCCTCTGC; exon 33 rev: AAACCCTGTAGGATTACCTTCTTGA were used. PCR products were separated as detailed above for genotyping PCRs.
