# Supplementary material for: Mouse superkiller‐2‐like helicase DDX60 is dispensable for type I IFN induction and immunity to multiple viruses
Source: Eur J Immunol. 2015 Oct 12;45(12):3386–403. doi: 10.1002/eji.201545794 (PMC4833184; doi:10.1002/eji.201545794)
Supplement: Supplementary file 1 — Supplementary Material [file eji3448-sup-0002-text.pdf › eji3448-sup-0002-text.pdf]

European Journal of Immunology

Supporting Information
for
DOI 10.1002/eji.201545794

Delphine Goubau, Annemarthe G. van der Veen, Probir Chakravarty, Rongtuan Lin, Neil Rogers, Jan Rehwinkel, Safia Deddouche, Ian Rosewell, John Hiscott and Caetano Reis e Sousa-C

Mouse superkiller-2-like helicase DDX60 is dispensable for type I IFN induction and immunity to multiple viruses

© 2015 The Authors. *European Journal of Immunology* published by WILEY-VCH Verlag GmbH & Co. KGaA, Weinheim.

European Journal of Immunology

Mouse superkiller-2-like helicase DDX60 is dispensable for type I IFN induction and immunity to multiple viruses

Gpubau, Delphine; Chakravarty, Probir; Lin, Rongtuan; Rogers, Neil; Rehwinkel, Jan; Deddouche, Safia; Rosewell, Ian; Hiscott, John; Reis e Sousa, Caetano

Correspondence: Prof. Reis e Sousa, Caetano, 44 Lincoln's Inn Fields, London, WC2A 3PX, Tel:+44 20 7269 2832

Review Timeline:	Submission date:	12-May-2015
	First Editorial decision:	01-Jun-2015
	First Revision received:	12-Aug-2015
	Accepted:	7-Sep-2015

Handling Executive Committee member: Prof. Annette Oxenius

Please note that the correspondence below does not include the standard editorial instructions regarding preparation and submission of revised manuscripts, only the scientific revisions requested and addressed.

First Editorial Decision - 01-Jun-2015

Dear Prof. Reis e Sousa,

Manuscript ID eji.201545794 entitled "Mouse superkiller-2-like helicase DDX60 is dispensable for type I IFN induction and immunity to multiple viruses" which you submitted to the European Journal of Immunology has been reviewed. The comments of the referees are included at the bottom of this letter.

We noticed that reviewer 3 asked for a lot of additional work in regard to the recent publication of Oshiumi et al.. We feel that your manuscript already contains a lot of data and we do not think that it is your job to identify the difference between your data and the data just published by Oshiumi because this publication has come out 2 weeks ago.

Therefore, out of the points of reviewer 3 we only ask you to:

1) Add one experiment with â€œshort poly(I:C)â€• stimulation in addition to in vitro transcribed short dsRNA (IVT-RNA) or standard poly(I:C).

2) Show that no truncated DDX60 ORF is expressed

A revised version of your manuscript that takes into account these comments and the comments of the other referees will be reconsidered for publication. Should you disagree with any of the refereesâ€™ concerns, you should address this in your point-by-point response and provide solid scientific reasons for why you will not make the requested changes.

You should also pay close attention to the editorial comments included below. **In particular, please edit your figure legends to follow Journal standards as outlined in the editorial comments. Failure to do this will result in delays in the re-review process.**

Please note that submitting a revision of your manuscript does not guarantee eventual acceptance, and that your revision will be re-reviewed by the referees before a decision is rendered.

If the revision of the paper is expected to take more than three months, please inform the editorial office. Revisions taking longer than six months may be assessed by new referees to ensure the relevance and timeliness of the data.

Once again, thank you for submitting your manuscript to European Journal of Immunology and we look forward to receiving your revision.

Yours sincerely,
Katharina Schmidt

On behalf of
Prof. Annette Oxenius

Dr. Katharina Schmidt
Editorial Office
European Journal of Immunology
e-mail: ejied@wiley.com
www.eji-journal.eu
****

Reviewer: 1

Comments to the Author
DDX60 was shown before to have a more narrow antiviral activity which affects HCV replication

(Schoggins et al Nature 472, 481â€“485; 2001). Another lab showed that DDX60 is invovled in the RIG-I signaling pathway, which suggests a broader antiviral activity (Oshiumi H et al Cell Rep. 2015 May 12. pii: S2211-1247(15)00464-7; Miyashita et al Mol. Cell. Biol. September 2011 vol. 31 no. 18 3802-3819).

Goubau et al. show now in their publication that while DDX60 is upregulated during virus infection, there is no influence of DDX60 on RIG-I signaling. Further DDX60 is not needed for protection after infection with several mouse viruses.

In pull down experiments Goubau et al. did not see interaction of DDX60 with RIG-I suggesting that at least in their systems this signaling interaction does not take place. Further, Goubau et used DDX60 deficient cells and animals and showed that absence of DDX60 does not lead to reduced induction of IFN-I after RIG-I activation. The experiments were performed in broad titrations, excluding dose dependency effects as explanation for the incoherence of their data with published observations. In line with these data survival experiments revealed no clear contribution of DDX60 to protection against different virus infections. These in vivo experiments where done with doses, which allows to detect small differences between DDX60 deficient and WT mice. In the discussion section, the authors discuss all biological and technical reasons for the discrepancy between their data and the so far published observations. They conclude their manuscript with the most likely explanation, namely, that DDX60 is a molecule with narrow antiviral activity.

Obviously it is always a challenge to prove that a molecule does not play an important role in certain situations. However the authors achieved with a tremendous set of data including titration experiments and multiple virus testing, that indeed DDX60 has not the broad antiviral activity as it is known for RIG-I. The data are very well performed and convincing. Because DDX60 is a new emerging innate molecule, this publication is very exciting for the field of immunology & virology. I recommend publications without any further revision.

Reviewer: 2

Comments to the Author
DDX60 has previously been proposed as both a viral restriction factor, and as a pattern recognition receptor (PRR) that cooperates with RIG-I-like receptors (RLRs) to sense viral nucleic acid. In fact numerous DDX proteins have been proposed to be involved in RNA and DNA sensing in many high impact publications in the past five years. However none of these DDX proteins have been tested for roles in innate immunity in vivo. Here the authors show that in contrast to previous work, in their hands DDX60

does not recognize viral RNA in vitro, nor does it cooperate with RLRs in interferon induction. Importantly they make two independent KO mice lacking DDX60 and demonstrate no role for DDX60 in RNA virus recognition in MEF and BMMC cells from these mice. Using these mice, they test the role of DDX60 in vivo in responses to influenza, EMCV, Sindbis virus, vaccinia and Listeria. In each case, responses are normal in the DDX60 KO mice. Although all the data in the paper are negative, this paper makes a very important clarifying contribution to the controversial area of the role of DDX proteins in innate immunity, and also suggests differences between human and mouse DDX60 in terms of anti-viral responses. Any role(s) for DDX60 in immunity in the mouse remain to be discovered.

Minor points:
1. The Discussion should mention that DDX60 may yet have roles in induction of cytokines not measured in this paper, and in primary mouse cell types yet to be tested.
2. Please also include discussion points addressing which DDX proteins, if any, apart from RLS, have been shown to date to have a role in innate sensing in vivo

Reviewer: 3

Comments to the Author
In this manuscript Goubau and colleagues report on the role of murine DDX60 in the context of antiviral immunity. In brief, their manuscript can be summarized as follows: Given its homology to yeast Ski2, a component involved in viral RNA degradation, the authors decided to study DDX60 in antiviral immunity (in the progress of their study a manuscript came out that reported a positive regulatory role of DDX60 in RLR signaling). First the authors show that DDX60 is broadly expressed and also IFN-inducible. Heterologous expression of human DDX60 or truncations thereof in HEK293 cells does not modulate RLR signaling. In line with these data, murine DDX60 KO cells (MEFs, macrophages) show no defects in response to synthetic RIG-I/MDA5 ligands or agonistic viruses. At the same time, DDX60 does not co-IP with RLRs, nor does it bind to RLR stimulatory ligands. Finally, DDX60-deficient mice show no defects in response to a wide array of live pathogens, including influenza virus. Based on these data the authors conclude that DDX60 doesnâ€™t play a major role in regulating RLR responses.

Up until recently, one publication in 2011 by Seya and colleagues has shown that DDX60 plays a positive regulatory role upstream of RLR signaling, yet no independent confirmation of this result has been provided (Moreover, in a screening approach for antiviral functions of ISGs by Rice and colleagues, DDX60 came up as a factor that negatively impacted on HCV replication upon overexpression, independently of RLRs). Given the fact that a number of solitary (and somewhat contradictory) reports

have implicated a number of RNA helicases in the context of nucleic acid sensing, the field has been somewhat hesitant to accept these molecules as bona fide PRRs or PRR modulators. However, just two weeks ago, the group of Seya has published another manuscript on the role of DDX60 in RLR signaling, in which data from a DDX60-deficient mouse have been provided. In contrast to the work provided by Goubau et al. this manuscript shows that DDX60 deficiency indeed has an impact on RIG-I signaling. Given the fact that similar experimental approaches were taken and similar stimuli were used, these discrepancies are quite puzzling. Nevertheless, there are some differences that might explain these discrepant results:

i) As synthetic stimuli Oshiumi et al. have mainly used â€œshort poly(I:C)â€• for their studies, whereas Goubau et al. used in vitro transcribed short dsRNA (IVT-RNA) or standard poly(I:C). While short poly(I:C) and in IVT-RNA both activate RIG-I, there might be differences with regards to their DDX60 agonistic activity.
I would suggest that the authors test short pol(I:C) as Oshiumi et al. to directly compare these results.

ii) While Oshiumi et al. excised the whole cassette located in between the loxP sites with out prior removal of the splice acceptor / lacZ cassette, Goubau et al. first removed the Frt-flanked insert and then crossed these mice with Cre deleter animals. Both configurations delete exon 9, whereas the â€œOshiumi alleleâ€• leaves a splice acceptor site, which additionally terminates transcription by a strong poly(A) signal. This could make a difference if the exon 9 deleted allele would still give rise to an alternatively spliced transcript that could still be translated. The authors allude to the possibility of NMD degrading the exon 9-less derived transcript(s), but these data arenâ€™t shown.
In light of the fact that no mouse-reactive DDX60 antibody is available, the authors need to rule out that a residual, truncated DDX60 ORF is expressed.

iii) Oshiumi et al. study two different in vivo models: hydrodynamic injection of HCV RNA and VSV (i.p. injection), in which DDX60 competence plays a positive role with regards to type I IFN production and also viral replication independently of type I IFN production. In their VSV model, there seems to be a considerable impact of MAVS-deficiency (yet unfortunately different infection conditions were used for different experiments). MAVS deficiency playing a role is an important pre-requisite to study the role of DDX60, which is supposed to be upstream of RLR-MAVS signaling. Goubau and colleagues study 5 viruses in the context of DDX60 deficiency without any major impact of DDX60. However, it is unclear whether RLR signaling indeed contributes to the antiviral immunity under these conditions (the EMCV data include a MAVS KO mouse, but it is unclear whether MAVS has a significant impact in this setting).
It would be of great interest, if the authors would at least test VSV in vitro and in vivo to compare their results directly to the study by Oshiumi and colleagues.

iv) Oshiumi et al. observe a profound differences in DDX60 requirement for different cell types. For example, bone marrow derived dendritic cells appear to signal independently of DDX60, whereas macrophages require DDX60. It is unclear how similar the macrophage / DC cell culture conditions of these two studies are (Goubau et al. for example use GM-CSF for their macrophage cultures, which Oshiumi et al. use for their DC cultures).
To harmonize settings, I would strongly recommend that the authors re-test the role of DDX60 in primary cell types directly isolated from the animals. For example, CD11c+ cells isolated from spleens should be of interest.

Altogether, the authors should provide experimental data that matches as much as possible with the settings described by Oshiumi et al., so that the reader can directly compare these studies. As outlined above, differences in ligands (synthetic and viruses), cell types, KO strategy and in vivo models currently preclude the conclusion of DDX60 being dispensable for RLR signaling. The study would be of a lot more interest, if a clear cut conclusion on the (non-)existing role of DDX60 in RLR signaling could be given. In light of the recent results by Oshiumi an colleagues, this is currently not the case.

Additional points:

- In many critical experiments (Fig 2, 3, 4 and 5) triplicate values + SD of technical replicates are shown. Given the importance of their negative results, the authors should show summarized data from several independent experiments and provide statistical analysis of their difference. Of note, a statistical test on technical replicates is not informative, as it only tells us how robust the measurement technology is (see Fig 4d).

-If a value is not detected (see Fig. 3), this should be indicated by n.d., instead of an asterisk.

<u>First Revision – authors' response – 12-Aug-2015</u>

Reviewer: 1
DDX60 was shown before to have a more narrow antiviral activity which affects HCV replication (Schoggins et al Nature 472, 481–485; 2001). Another lab showed that DDX60 is invovled in the RIG-I signaling pathway, which suggests a broader antiviral activity (Oshiumi H et al Cell Rep. 2015 May 12. pii: S2211-1247(15)00464-7; Miyashita et al Mol. Cell. Biol. September 2011 vol. 31 no. 18 3802-3819).

Goubau et al. show now in their publication that while DDX60 is upregulated during virus infection, there is no influence of DDX60 on RIG-I signaling. Further DDX60 is not needed for protection after infection with several mouse viruses.

In pull down experiments Goubau et al. did not see interaction of DDX60 with RIG-I suggesting that at least in their systems this signaling interaction does not take place. Further, Goubau et used DDX60 deficient cells and animals and showed that absence of DDX60 does not lead to reduced induction of IFN-I after RIG-I activation. The experiments were performed in broad titrations, excluding dose dependency effects as explanation for the incoherence of their data with published observations. In line with these data survival experiments revealed no clear contribution of DDX60 to protection against different virus infections. These in vivo experiments where done with doses, which allows to detect small differences between DDX60 deficient and WT mice. In the discussion section, the authors discuss all biological and technical reasons for the discrepancy between their data and the so far published observations. They conclude their manuscript with the most likely explanation, namely, that DDX60 is a molecule with narrow antiviral activity.

Obviously it is always a challenge to prove that a molecule does not play an important role in certain situations. However the authors achieved with a tremendous set of data including titration experiments and multiple virus testing, that indeed DDX60 has not the broad antiviral activity as it is known for RIG-I. The data are very well performed and convincing. Because DDX60 is a new emerging innate molecule, this publication is very exciting for the field of immunology & virology. I recommend publications without any further revision.

We thank the reviewer for his/her comments and endorsement of our manuscript.

Reviewer: 2
DDX60 has previously been proposed as both a viral restriction factor, and as a pattern recognition receptor (PRR) that cooperates with RIG-I-like receptors (RLRs) to sense viral nucleic acid. In fact numerous DDX proteins have been proposed to be involved in RNA and DNA sensing in many high impact publications in the past five years. However none of these DDX proteins have been tested for roles in innate immunity in vivo. Here the authors show that in contrast to previous work, in their hands DDX60 does not recognize viral RNA in vitro, nor does it cooperate with RLRs in interferon induction. Importantly they make two independent KO mice lacking DDX60 and demonstrate no role for DDX60 in RNA virus recognition in MEF and BMMC cells from these mice. Using these mice, they test the role of DDX60 in vivo in responses to influenza, EMCV, Sindbis virus, vaccinia and Listeria. In each case, responses are normal in the DDX60 KO mice. Although all the data in the paper are negative, this paper makes a very important clarifying contribution to the controversial area of the role of DDX proteins in innate immunity,

and also suggests differences between human and mouse DDX60 in terms of anti-viral responses. Any role(s) for DDX60 in immunity in the mouse remain to be discovered.

We thank the reviewer for his/her comments and endorsement of our manuscript.

Minor points:
1. The Discussion should mention that DDX60 may yet have roles in induction of cytokines not measured in this paper, and in primary mouse cell types yet to be tested.

We thank the reviewer for this suggestion and have included this as a discussion point.

2. Please also include discussion points addressing which DDX proteins, if any, apart from RLS, have been shown to date to have a role in innate sensing in vivo

This point has now been included in the introduction to the manuscript.

Reviewer: 3
In this manuscript Goubau and colleagues report on the role of murine DDX60 in the context of antiviral immunity. In brief, their manuscript can be summarized as follows: Given its homology to yeast Ski2, a component involved in viral RNA degradation, the authors decided to study DDX60 in antiviral immunity (in the progress of their study a manuscript came out that reported a positive regulatory role of DDX60 in RLR signaling). First the authors show that DDX60 is broadly expressed and also IFN-inducible. Heterologous expression of human DDX60 or truncations thereof in HEK293 cells does not modulate RLR signaling. In line with these data, murine DDX60 KO cells (MEFs, macrophages) show no defects in response to synthetic RIG-I/MDA5 ligands or agonistic viruses. At the same time, DDX60 does not co-IP with RLRs, nor does it bind to RLR stimulatory ligands. Finally, DDX60-deficient mice show no defects in response to a wide array of live pathogens, including influenza virus. Based on these data the authors conclude that DDX60 doesn't play a major role in regulating RLR responses.

Up until recently, one publication in 2011 by Seya and colleagues has shown that DDX60 plays a positive regulatory role upstream of RLR signaling, yet no independent confirmation of this result has been provided (Moreover, in a screening approach for antiviral functions of ISGs by Rice and colleagues, DDX60 came up as a factor that negatively impacted on HCV replication upon overexpression, independently of RLRs). Given the fact that a number of solitary (and somewhat contradictory) reports have implicated a number of RNA helicases in the context of nucleic acid sensing, the field has been somewhat hesitant to accept these molecules as bona fide PRRs or PRR modulators. However, just two weeks ago, the group of Seya has published another manuscript on the role of DDX60 in RLR signaling, in

European Journal of
Immunology

which data from a DDX60-deficient mouse have been provided. In contrast to the work provided by Goubau et al. this manuscript shows that DDX60 deficiency indeed has an impact on RIG-I signaling. Given the fact that similar experimental approaches were taken and similar stimuli were used, these discrepancies are quite puzzling. Nevertheless, there are some differences that might explain these discrepant results:

We thank the reviewer for his/her comments. We were equally puzzled by the very recent report from Oshiumi et al., which was published subsequent to our submission to EJI. Indeed, they suggest in a mouse model very similar to ours that DDX60-deficiency in certain cell types attenuates RIG-I signalling.

i) As synthetic stimuli Oshiumi et al. have mainly used “short poly(I:C)” for their studies, whereas Goubau et al. used in vitro transcribed short dsRNA (IVT-RNA) or standard poly(I:C). While short poly(I:C) and in IVT-RNA both activate RIG-I, there might be differences with regards to their DDX60 agonistic activity.
I would suggest that the authors test short pol(I:C) as Oshiumi et al. to directly compare these results.

As suggested by the reviewer, we tested short poly(I:C) in parallel to standard poly(I:C) in our DDX60 KO model. As for Oshiumi et al. (Figure 1E), we used MEFs and analysed IFN-□ levels. In our hands, similarly to standard poly(I:C), DDX60 deficiency did not impact the response to low molecular weight short poly(I:C) in primary or immortalized MEFs. The data are now included in Figure 4A.

We believe that these data allow greater comparison between our work and that of Oshiumi et al. even if they do not resolve the discrepancy between the two studies. The differences in our manuscripts are not limited to the results obtained using KO cells as we also do not observe any interaction between human DDX60, other RLRs and nucleic acids. Given the profound differences in our results, we believe that instead of trying to recreate the exact conditions in which Seya’s group have found a role for DDX60 in RLR-mediated signaling, we should publish our findings ASAP so that independent third party investigations of the role of DDX60 in innate immunity can be conducted.

ii) While Oshiumi et al. excised the whole cassette located in between the loxP sites with out prior removal of the splice acceptor / lacZ cassette, Goubau et al. first removed the Frt-flanked insert and then crossed these mice with Cre deleter animals. Both configurations delete exon 9, whereas the “Oshiumi allele” leaves a splice acceptor site, which additionally terminates transcription by a strong poly(A) signal. This could make a difference if the exon 9 deleted allele would still give rise to an alternatively spliced transcript that could still be translated. The authors allude to the possibility of NMD degrading the exon 9-less derived transcript(s), but these data aren’t shown.
In light of the fact that no mouse-reactive DDX60 antibody is available, the authors need to rule out that a

residual, truncated DDX60 ORF is expressed.

We have now included data in Supportive Figure S2C showing the lack of amplification of exons 29 to 36 when cDNA from DDX60 full-KO cells is used. These results are consistent with the interpretation that exon 9 -deleted DDX60 transcripts are degraded by NMD. We also conducted more extensive PCR analysis in DDX60-full KO and DDX60 WT cells and did not observe any additional truncated DDX60 ORFs. These data are now included in Supportive Figure S2D.

iii) Oshiumi et al. study two different in vivo models: hydrodynamic injection of HCV RNA and VSV (i.p. injection), in which DDX60 competence plays a positive role with regards to type I IFN production and also viral replication independently of type I IFN production. In their VSV model, there seems to be a considerable impact of MAVS-deficiency (yet unfortunately different infection conditions were used for different experiments). MAVS deficiency playing a role is an important pre-requisite to study the role of DDX60, which is supposed to be upstream of RLR-MAVS signaling. Goubau and colleagues study 5 viruses in the context of DDX60 deficiency without any major impact of DDX60. However, it is unclear whether RLR signaling indeed contributes to the antiviral immunity under these conditions (the EMCV data include a MAVS KO mouse, but it is unclear whether MAVS has a significant impact in this setting).
It would be of great interest, if the authors would at least test VSV in vitro and in vivo to compare their results directly to the study by Oshiumi and colleagues.

The use of VSV is highly regulated in the United Kingdom due to the ability of the virus to infect horses, cattle and pigs with serious economic and commercial losses. We have previously attempted to import VSV for use in our laboratory but were unable to do so due to the severe restrictions. As such, we cannot perform the requested experiment.

iv) Oshiumi et al. observe a profound differences in DDX60 requirement for different cell types. For example, bone marrow derived dendritic cells appear to signal independently of DDX60, whereas macrophages require DDX60. It is unclear how similar the macrophage / DC cell culture conditions of these two studies are (Goubau et al. for example use GM-CSF for their macrophage cultures, which Oshiumi et al. use for their DC cultures).
To harmonize settings, I would strongly recommend that the authors re-test the role of DDX60 in primary cell types directly isolated from the animals. For example, CD11c+ cells isolated from spleens should be of interest.

As we include in the Discussion of our manuscript, we agree that some of the differences observed in our studies may partly lie in the different cell types used (primary vs. transformed; bone-marrow -derived cells versus peritoneal macrophages). However, as agreed with the editors, we do not believe that the onus

should be on us to systematically examine every different cell type used by Oshiumi et al., especially as we were not aware of their manuscript when ours was submitted to EJI. As such, we would suggest that our manuscript be published without further delay to allow the community to compare the two studies.

Altogether, the authors should provide experimental data that matches as much as possible with the settings described by Oshiumi et al., so that the reader can directly compare these studies. As outlined above, differences in ligands (synthetic and viruses), cell types, KO strategy and in vivo models currently preclude the conclusion of DDX60 being dispensable for RLR signaling. The study would be of a lot more interest, if a clear cut conclusion on the (non-)existing role of DDX60 in RLR signaling could be given. In light of the recent results by Oshiumi an colleagues, this is currently not the case.

We respectfully disagree with the reviewer's comment and believe that although our paper presents only negative data, in light of the two current manuscripts published on DDX60 describing a role for this helicase in RIG-I-dependent responses, our study stands on its own merit and is of great interest to the community.

Additional points:
- In many critical experiments (Fig 2, 3, 4 and 5) triplicate values + SD of technical replicates are shown. Given the importance of their negative results, the authors should show summarized data from several independent experiments and provide statistical analysis of their difference. Of note, a statistical test on technical replicates is not informative, as it only tells us how robust the measurement technology is (see Fig 4d).

The majority of experiments presented here were performed at least three times. We have chosen to show one representative experiment. Although we agree with the reviewer in that, in principle, pooling data from three independent experiments can in some cases be beneficial, practically, this is not always possible with the assays conducted as often the absolute magnitude of the response readout can vary. We are confident of the data presented as, in addition to having repeated the experiments multiple times, we have included dose responses for every stimulus used.

We have removed the statistical analysis made on technical replicates.

-If a value is not detected (see Fig. 3), this should be indicated by n.d., instead of an asterisk.
This has been modified.

European Journal of Immunology

Second Editorial Decision - 20-Aug-2015

Dear Prof. Reis e Sousa,

It is a pleasure to provisionally accept your manuscript entitled "Mouse superkiller-2-like helicase DDX60 is dispensable for type I IFN induction and immunity to multiple viruses" for publication in the European Journal of Immunology. For final acceptance, please follow the instructions below and return the requested items as soon as possible as we cannot process your manuscript further until all items listed below are dealt with.

Please note that EJI articles are now published online a few days after final acceptance (see Accepted Articles: http://onlinelibrary.wiley.com/journal/10.1002/(ISSN)1521-4141/accepted). The files used for the Accepted Articles are the final files and information supplied by you in Manuscript Central. You should therefore check that all the information (including author names) is correct as changes will NOT be permitted until the proofs stage.

We look forward to hearing from you and thank you for submitting your manuscript to the European Journal of Immunology.

Yours sincerely,
Katharina Schmidt

on behalf of
Prof. Annette Oxenius

Dr. Katharina Schmidt
Editorial Office
European Journal of Immunology
e-mail: ejied@wiley.com
www.eji-journal.eu
